# Enhancing Prime Editing Efficiency Through Modulation of Methylation on the Newly Synthesized DNA Strand and Prolonged Expression

**DOI:** 10.1002/advs.202417790

**Published:** 2025-03-07

**Authors:** Xiaosong Han, Xianghua Xu, Youcai Xiong, Guangxing Zhao, Ruigao He, Yinyu Su, Sheng Li, Changzhi Zhao, Xiaoning Xi, Yunxia Zhao, Xuewen Xu, Shengsong Xie, Heng Wang, Xinyun Li, Shuhong Zhao, Jinxue Ruan

**Affiliations:** ^1^ Key Laboratory of Agricultural Animal Genetics Breeding and Reproduction of Ministry of Education & Key Laboratory of Swine Genetics and Breeding of Ministry of Agriculture and Rural Affairs Huazhong Agricultural University Wuhan 430070 P. R. China; ^2^ Yazhouwan National Laboratory Sanya 572024 P. R. China; ^3^ Frontiers Science Center for Animal Breeding and Sustainable Production Huazhong Agricultural University Wuhan 430070 P. R. China; ^4^ Hubei Hongshan Laboratory Frontiers Science Center for Animal Breeding and Sustainable Production Wuhan 430070 P. R. China

**Keywords:** EBNA1/oriP, gene editing, methylation, multiplex, prime editor

## Abstract

Prime editors (PEs) have emerged as transformative tools for precision genome engineering, yet their broader application remains constrained by incomplete understanding of repair mechanisms. In this study, it is found that an increase in the methylation level of the CpG sequence on the newly generated strand can increase PE efficiency and that *de novo* DNA methyltransferases (DNMT3A/3B) are involved in the PE repair pathway. On the basis of these novel findings, the development of an episomal element‐driven PE system (epiPE) achieved through the use of EBNA1/oriP are presented, which increases methylation levels around target sites and prolongs PE expression. A comparative analysis with canonical PE systems, including PE2, lentiPE2, and PE4max, reveals that the epiPE2 system significantly enhances editing efficiency while maintaining minimal insertion and deletion (indels) rates. Specifically, comparing to PE2, the epiPE2 system demonstrated an efficiency enhancement of 2.0 to 38.2‐fold. In addition, the epiPE2 system is capable of efficient multiplex precise gene editing at up to 10 genetic loci in human cells. In conclusion, this findings increase the understanding of the PE repair mechanism, and presents the epiPE2 system as an efficient and multiplex‐capable prime editing tool with potential applications in both basic research and translational studies.

## Graphical abstract

Prime editors (PEs) enhance gene editing precision but face repair mechanism limitations. DNA methylation (CpG sites) and *de novo* methyltransferases (DNMT3A/DNMT3B) boost PE efficiency. We developed an episomal PE system (epiPE) using EBNA1/oriP to sustain expression and methylation. EpiPE2 outperformed PE2 (2.0–38.2‐fold efficiency gain) with minimal indels and enabled multiplex editing (10 loci). Findings advance PE mechanistic insights and therapeutic applications.

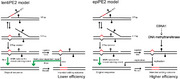

## 1 Introduction

Programmable genome‐editing tools have shown therapeutic potential for genetic disorders.^[^ ^1^, ^2^, ^3^, ^4^, ^5^, ^6^ ^]^ Prime editors (PEs) generate minimal double‐strand breaks (DSBs), can install various targeted DNA base pair substitutions and small insertions and deletions, without the need to co‐transfer donor templates.^[^ ^6^, ^7^, ^8^ ^]^ PE2, a representative canonical PE variant, consists of a Cas9 nickase (H840A) fused with an engineered reverse transcriptase (RTase) and a prime editing guide RNA (pegRNA) that contains a single guide RNA (sgRNA) sequence complementary to the target genome DNA at the 5′ end and a 3′ extension encoding the desired edits.^[^ ^7^ ^]^ Nevertheless, editing efficiency varies across different cell types and species, and the mechanisms underlying PEs are not yet well understood, which hampers the development and application of the PE system, despite numerous recent efforts to advance it.

Several studies have reported that the mismatch repair (MMR) pathway strongly impairs PE editing efficiency by limiting the desired outcomes during the prime editing process.^[^ ^8^, ^9^ ^]^ The key premise of the MMR is how to distinguish between parental (old and correct) and daughter (new but may contain errors) strands. Therefore, the desired edits that form mismatches tend to be repaired/removed by MMR during the PE process, greatly reducing editing efficiency. Realizing this, PE4/PE5 was developed by harnessing a dominant‐negative variant of an MMR protein, MLH1dn, to inhibit MMR activity as a means to promote PE efficiency.^[^ ^8^ ^]^

Furthermore, previous studies have suggested that cytosine methylation may play a role in strand discrimination in mammalian cells.^[^ ^10^, ^11^ ^]^ Recent evidence suggests that cytosine methylation of CpGs and DNA methyltransferases is involved in the MMR pathway and has an impact on strand discrimination in eukaryotes.^[^ ^12^, ^13^, ^14^ ^]^ On this basis, we hypothesized that in the PE process, the newly synthesized strand containing the desired edits, without methylation, is likely regarded as the “wrong strand” by MMR and is subsequently “repaired”, leading to a decrease in PE efficiency. Therefore, we hypothesize that the PE efficiency can be improved if the newly synthesized strand is quickly methylated, thus avoiding the MMR process.

In the present study, we identified that the episomal elements EBNA1/oriP as an effective solution to the aforementioned strategies. The EBNA1/oriP elements consist of two key components: oriP, the viral origin of replication, and EBNA1, the Epstein‐Barr nuclear antigen 1 protein. EBNA1 binds to specific DNA sequences within oriP, a critical step for oriP‐dependent DNA replication and the maintenance of episomes. In addition to facilitating the recruitment of cellular factors such as the origin recognition complex and minichromosome maintenance (MCM) proteins to initiate replication, EBNA1 also plays a key role in stabilizing the episomes. Importantly, studies indicate that EBNA1 can enhance the expression of intracellular methyltransferases and increase the methylation levels of genes.^[^ ^15^ ^]^

Here we constructed epiPE2 by including EBNA1/oriP in the PE2 system. Compared with canonical PE2 (cPE2), epiPE2 enhances the precise editing efficiency by 2.0‐ to 38.2‐fold in HEK293T cells while maintaining a very low indel frequency comparable to those of cPE2. Compared with the later PE versions, which include PE3 and PE4max, epiPE2 also exhibited greater editing efficiency across all the tested loci. Mechanistically, we demonstrated that epiPE2 improves editing efficiency by increasing the methylation level of the CpG sequence on the newly generated strand and by prolonging the expression duration of PE2 components in cells. We show that the *de novo* DNA methyltransferases DNMT3A and DNMT3B participate in the PE repair process. In addition, we also demonstrated the efficient multiplex editing capacity of epiPE2, which targets up to 10 loci simultaneously, making it a potential tool for treating complex genomics and polygenic diseases.

## 2 Results

### 2.1 The epiPE2 System for Prime Editing

**Figure 1:**
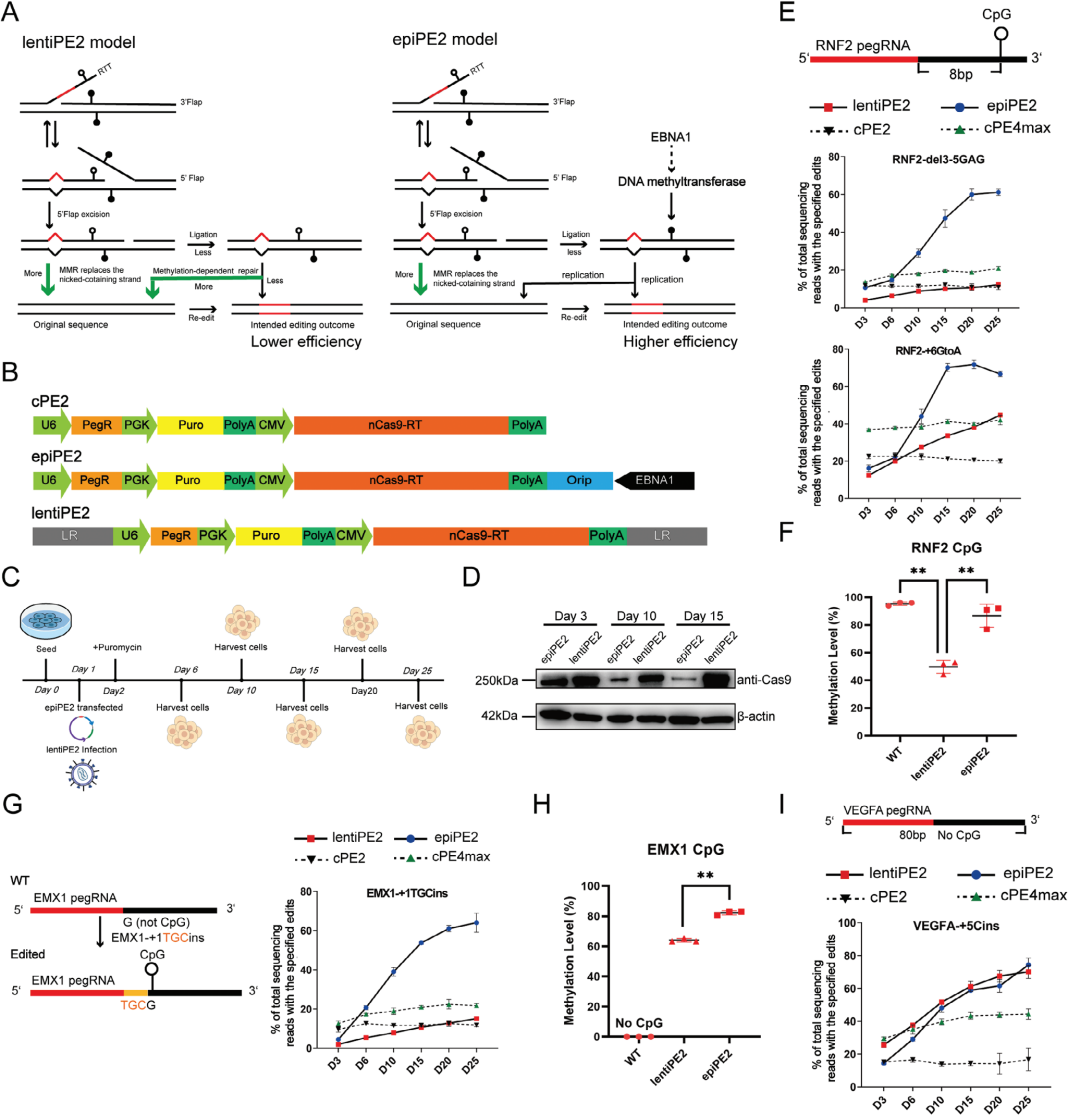
epiPE2 increases PE2 editing efficiency by increasing the methylation level of CpGs around target sites. A) Repair models for the lentiPE2 (left) and epiPE2 (right) systems. During the repair process, MMR replaces the nick‐containing strand, resulting in a low intended editing efficiency, and we hypothesize that the PE editing process decreases the methylation level of the target region. B) Schematic diagrams of the cPE2, epiPE2 and lentiPE2 constructs. C) Workflow of the time points for the treatment of cells with the cPE2, epiPE2 and lentiPE2 systems. D) Western blot analysis of the PE protein expression levels in epiPE2‐ and lentiPE2‐treated cells on Days 3, 10, and 15 posttransfection and infection. E) Two pegRNAs were tested, and the editing efficiencies of the epiPE2, cPE2, cPE4max and lentiPE2 systems on the RNF2 locus were compared in HEK293T cells on Days 3, 6, 10, 15, 20, and 25. The upper panel shows the distance between the pegRNA and the CpG site. F) The methylation level of the CpG in the 5′ end of the pegRNA in the RNF2 locus used in e was analyzed and compared between epiPE2‐ and lentiPE2‐treated HEK293T cells. G) Editing efficiency was compared between the epiPE2 and lentiPE2 systems at the EMX1 locus in HEK293T cells on Days 3, 6, 10, 15, 20, and 25. The upper panel shows the distance between the pegRNA and the CpG site. H) The CpG methylation levels at the 5′ end of the pegRNA at the EMX1 locus used in g were analyzed and compared between epiPE2‐ and lentiPE2‐treated HEK293T cells. I) Editing efficiencies were compared between the epiPE2 and lentiPE2 systems at the VEGFA locus in HEK293T cells on Days 3, 6, 10, 15, 20 and 25. The upper panel shows that no CpG exists around the pegRNA used at this site. *P < 0.05, **P < 0.01, ***P < 0.001.

The EBNA1/oriP element, which originates from the Epstein‒Barr virus, has been previously reported to activate *de novo* DNA methyltransferases in vivo and was incorporated here into the PE2 system to generate the epiPE2 system.^[^ ^16^, ^17^ ^]^ The rationale behind this strategy was to quickly add methylation marks to the prime edited strand, which would prevent the endogenous MMR mechanism from recognizing it as a newly synthesized erroneous strand, thereby increasing the overall PE efficiency. In addition, EBNA1 episomal components are known to promote plasmid duplication per cell cycle (Figure S1A, Supporting Information) to achieve high expression of transgenes in mammalian cells.^[^ ^15^, ^18^, ^19^ ^]^ Thus, the introduction of EBNA1 to the PE2 system hypothetically results in two benefits: (i) reduced MMR repair of the PE edited strand, which increases PE editing rates, and (ii) an extended expression time leading to increased PE efficiency. **Figure** **1**A illustrates the design of epiPE2. The all‐in‐one epiPE2 vector comprised the pegRNA expression element, the PE2 expression element, a puromycin resistance expression cassette and the EBNA1/oriP fragment (Figure 1B and Experimental Section).

#### 2.1.1 epiPE2 Improves the Expression of PE Components

To verify whether epiPE can enhance the editing efficiency of the PE system, we first tested whether epiPE can improve the expression patterns of PE components. To achieve this goal, we transfected HEK293T cells with cPE2 and epiPE2 plasmids and evaluated the temporal expression patterns of PE2 (Figure 1C). We found that the expression level of epiPE2 remained stable and persistent for at least 25 days, while the cPE2 protein level was highest on Day 3 and rapidly decreased on Day 6, becoming undetectable thereafter (Figure S1B, Supporting Information). Furthermore, when puromycin was withdrawn on Day 25 post transfection of epiPE2, PE2 expression gradually decreased, indicating that the epiPE2 system is reversible (Figure S2, Supporting Information), which is consistent with previous studies.^[^ ^18^, ^20^, ^21^ ^]^

#### 2.1.2 epiPE2 Increases Editing Efficiency

We then compared the editing efficiency of the epiPE2 system with other PE systems in targeting the RNF2 locus in HEK293T cells. We selected cPE2 and cPE4max to represent canonical PE systems. Given that lentiviral vectors can also prolong the duration of PE protein expression, we constructed a lentiviral‐based PE system (lentiPE) and generated a HEK293T cell line with stable expression of the PE protein via infection with lentiPE. PE expression at both the transcriptional and translational levels between the epiPE2 and lentiPE2 systems revealed that the mRNA expression level of PE in the lentiPE system was greater than that in the epiPE system across a period of 25 days (Figure S3, Supporting Information), and the overall levels of PE proteins were greater in the lentiPE2 system than in the epiPE2 system (Figure 1D).

The results revealed that, compared with the cPE2 and cPE4max systems, the epiPE2 system presented the highest editing efficiency. Interestingly, although the expression of the PE element by epiPE2 was lower than that of the lentiPE2 system, the epiPE2 editing efficiency remained consistently much greater than that of the lentiPE2 system at the tested loci throughout the experimental course, from Day 3 to Day 25 (Figure 1E,G). These results suggest that epiPE can increase PE efficiency and that factors other than the expression level of the PE protein may have affected PE efficiency. Specifically, we hypothesized that the episomal elements in epiPE2 might promote efficiency; Thus, we transfected EBNA1/oriP‐containing plasmids into HEK293T cells 6 days after lentiPE2 infection. The efficiency was evaluated on Day 10 after lentiPE2 infection (Figure S4, Supporting Information) and was improved compared with that in the control group, indicating that episomal elements played a role other than only promoting expression.

#### 2.1.3 epiPE2 Increases Editing Efficiency by Promoting the Methylation of CpG on Newly Synthesized DNA Strands

Because episomal elements can upregulate the expression of *de novo* DNA methyltransferase,^[^ ^16^, ^17^ ^]^ we hypothesized that epiPE2, which contains these episomal elements, may upregulate DNA methylation at the target locus and subsequently promote PE2‐mediated gene editing. Thus, we performed qPCR to compare the transcription levels of the DNMT3A and DNMT3B genes between cPE2‐ and epiPE2‐transfected cells. The results revealed that the mRNA expression levels of DNMT3A and DNMT3B were significantly increased in both the cPE2 and epiPE2 contexts (Figure S5, Supporting Information) in comparison to the non‐transfected cells, but the increases were greater in the epiPE2 system than in the cPE2 system. Of note, the level of increase of DNMT3A and DNMT3B transcriptions is positively correlated with the PE rates achieved by epiPE2 and cPE2 (Figure S5, Supporting Information).

We compared the methylation levels of a CpG sequence proximal to the pegRNA target locus at RNF2 between epiPE2‐ and lentiPE2‐edited cells. Using bisulfite treatment of genomic DNA followed by amplicon sequencing, we found that compared with lentiPE2‐treated cells, epiPE2‐treated cells had higher methylation levels of CpG in the target proximate region (Figure 1F). Our findings suggest that the presence of a CpG methylation site adjacent to the pegRNA target sequence and the extent of CpG methylation may affect editing efficiency.

To investigate whether the presence and the extent of CpG methylation could promote PE efficiency, we designed another pegRNA targeting the EMX1 locus, which creates a CG sequence after successful PE editing. On the basis of our hypothesis, we predicted that this new CpG sequence would be methylated after epiPE2 editing, leading to high PE editing rates; In contrast, lentiPE2 would not methylate this new CpG sequence and would have low editing efficiency. Indeed, our results concerning both the PE editing rates and the levels of methylation post editing in epiPE2‐ and lentiPE2‐edited cells supported our prediction (Figure 1G,H). Following the same rationale, we designed a pegRNA targeting the VEGFA locus, which lacks a CpG downstream. Consistent with our hypothesis, this pegRNA resulted in comparable editing rates in both epiPE2‐ and lentiPE2‐edited cells, and the methylation levels were low at both sites (Figure 1I). These results indicate that the epiPE2 system enhances the efficiency of PE editing by increasing the methylation level of CpGs downstream of the pegRNA.

### 2.2 PE2 Mediated Editing Reduces Methylation Levels of the Edited Strand

One other interesting finding we observed is that the methylation level around the targeted region in lentiPE2‐treated cells was significantly lower than in control cells. This suggests that the PE process reduces the methylation of the editing strand, in line with our hypothesis (Figure 1F).

To test this and to exclude potential influences from the epiPE system, we constructed an mCherry reporter system. In this system, the mCherry coding sequence contains an internal stop codon. Successful repair excises the stop codon, thereby activating red fluorescence. To facilitate cell enrichment, a GFP gene was also included in the vector. This mCherry reporter system was integrated into HEK293T cells, from which a monoclonal cell line was established (Figure S6A, Supporting Information). The mCherry‐stop reporter cell line was then transfected with the cPE2 vector targeting the internal stop codon.

mCherry‐positive cells (i.e., successfully PE edited) were collected at 0, 12, 24, and 36 hours post‐transfection, and genomic DNA was extracted to assess the methylation levels of the edited strand. The results showed that between 12 and 36 hours post‐transfection, methylation levels at this site were significantly lower than in mCherry‐positive cells than in mCherry‐negative cells (Figure S6B, Supporting Information). These results demonstrate that canonical PE reduces methylation levels of the edited strand, and support our strategy to modulate the methylation levels to promote PE efficiency.

#### 2.2.1 epiPE2 Mediated Methylation Occurs Earlier than MMR Repair

To gain more insights onto the methylation process in PE achieved by epiPE2 versus cPE2, we conducted experiments to check the methylation of the newly synthesized strand at different time points. Therefore, we selected 12, 24, 36, and 48‐hour time points to analyze methylation levels at the DNMT1 locus in K562 cells using both the epiPE2 and cPE2 systems. Our results show that as early as at 12 hours all the way through 36‐ and 48‐hours post transfection, the epiPE2 group had significantly higher methylation levels on the newly synthesized strand compared to the cPE2 group (Figure S7A, Supporting Information), suggesting that the methylation effect of epiPE2 starts before 12 hours and lasts for at least 48 hours. Based on previous studies, the plasmid expression time is around 6–12 hours, and dCas9 binding to the genomic DNA target happens around the same time.^[^ ^22^ ^]^ Our results therefore suggest that epiPE2 mediated methylation occurs before MMR repair (Figure S7B, Supporting Information).

### 2.3 Inhibition of DNA Methyltransferase Reduces PE Efficiency

**Figure 2:**
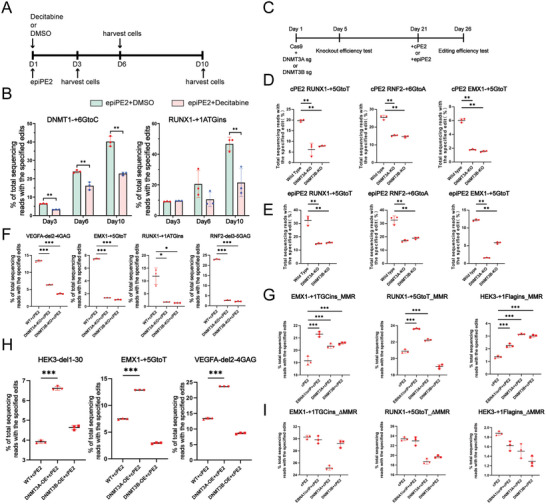
epiPE2 enhances PE2 editing efficiency via methyltransferases. A) Experimental workflow for decitabine‐ and DMSO‐treated cells. B) Comparison of the editing efficiencies between decitabine‐ and DMSO‐treated cells transfected with epiPE2. Frequencies (means ± s.e.m.) were calculated from two or three independent experiments. C) Experimental workflow for assessing the role of DNMT3A or DNMT3B in the PE2 editing system. D) Editing efficiency test of cPE2 in DNMT3A‐ and DNMT3B‐knockout cell pools. E) Editing efficiency test of epiPE2 in DNMT3A‐ and DNMT3B‐knockout cell pools. F) Editing efficiency test of cPE2 in DNMT3A‐ and DNMT3B‐knockout cell lines. G) The impact of EBNA1/oriP, DNMT3A and DNMT3B on PE editing efficiency in MMR‐proficient cells. H) Editing efficiency test of cPE2 in DNMT3A‐ and DNMT3B‐overexpressing cell lines. Frequencies (means ± s.e.m.) were calculated from two or three independent experiments. I) The impact of EBNA1/oriP, DNMT3A and DNMT3B on PE editing efficiency in MMR‐deficient cells. *P < 0.05, **P < 0.01, ***P < 0.001.

To further investigate whether the greater efficiency observed with epiPE2 is due to its methylation capacity, we transfected HEK293T cells with the epiPE2 plasmid supplemented with the DNA methyltransferase inhibitor decitabine. We collected epiPE2‐treated cells 3, 6, and 10 days after transfection and assessed their efficiency (**Figure** **2**A; Figure S8, Supporting Information). Our results demonstrated that, in comparison with those in the DMSO‐treated control group, the editing efficiencies at both tested loci significantly decreased in the decitabine‐treated group, further supporting the notion that the ability of epiPE2 to promote DNA methylation plays a critical role in enhancing PE2 editing efficiency (Figure 2B).

To further confirm this finding, we conducted the same experiment in MMR‐proficient K562 cells. The results showed that, for all three loci tested, the editing efficiency of both epiPE2 and cPE2 was significantly reduced upon inhibitor decitabine treatment (Supplementary Figure S9, Supporting Information).

To further investigate the potential role of DNMT3A and DNMT3B in PE systems, we generated pools of DNMT3A‐ and DNMT3B‐knockout cells. The knockout efficiencies were 73% and 92%, respectively (Figure S10A, Supporting Information). After culturing for more than 2 weeks, to ensure that the DNMT3A‐ and DNMT3B‐ targeting sgRNA/Cas9 plasmid are degraded, we transfected these cells with the cPE2 or epiPE2 elements (Figure 2C). Compared with those in the DNMT3A and DNMT3B wild‐type groups, the editing efficiencies of cPE2 and epiPE2 were significantly lower in both the DNMT3A and DNMT3B knockout groups (Figure 2D,E). To further substantiate the roles of DNMT3A and DNMT3B, we selected DNMT3A‐ and DNMT3B‐knockout cell clones and separately transfected them with cPE2. Again, the results revealed notable reductions in PE editing efficiency in both the DNMT3A‐ and the DNMT3B‐knockout cell clones relative to the wild‐type groups (Figure 2F; Figure S10B, Supporting Information). These findings provide additional evidence that DNMT3A and DNMT3B contribute to the PE2 editing system.

### 2.4 Overexpression of DNA Methylation Promoting Factors Improves PE Efficiency through Modulating the MMR Pathway

Next, we conducted an experiment in which the cells were transfected cPE2 along with one of the following: (i) EBNA1/oriP; (ii) DNMT3A and (iii) DNMT3B plasmids and into HEK293T cells, whereas the control group received only the corresponding cPE2 plasmids. The efficiency of the editing process was evaluated following the transfection of cells for a period of five days. The results demonstrated a significant improvement in PE2 editing efficiency, specifically at the HEK3 site, particularly with flag insertion (Figure 2G), was achieved co‐transfecting any of these three: EBNA1/oriP, DNMT3A, or DNMT3B.

To further substantiate the positive effects of DNMT3A and DNMT3B, we generated stable DNMT3A and DNMT3B expression HEK293T cell lines (named as DNMT3A‐OE and DNMT3B‐OE cell lines, respectively). The cells were subsequently transfected with cPE2. Compared with those in the wild‐type group, the editing efficiencies at three loci were significantly greater in the DNMT3A‐overexpressing cell line, exhibiting approximately 1.7‐fold greater efficiencies (Figure 2H). Taken together, these results provide further evidence of the contributions of EBNA1/oriP, DNMT3A, and DNMT3B to the PE2 editing system.

To investigate whether this enhancement is mediated through the MMR pathway, we generated MMR‐deficient cells by knocking out MLH1 in HEK293T cells, as MLH1 is a key protein in the MMR repair pathway. We then overexpressed EBNA1, DNMT3A, and DNMT3B in these cells and performed PE editing at different loci including RUNX1, EMX1 and HEK3. The results showed that EBNA1/DNMT3A/DNMT3B associated PE efficiency improves were abolished in the MMR‐deficient cells (Figure 2I). These findings suggest that EBNA1, DNMT3A, and DNMT3B likely enhance PE editing efficiency through modulating the MMR pathway.

### 2.5 Effects of CpG Position on epiPE2 Editing Efficiency

To determine the optimal distance between methylation sites and the pegRNA target that enhances PE efficiency, we conducted experiments using the HEK3 site. CpG sites were introduced at distances of 1, 10, 19, and 30 base pairs from the cleavage site, and editing efficiencies of epiPE2 and lentiPE2 were compared. The results indicated that epiPE2 outperformed lentiPE2 in editing efficiency within the 1 to 19 base pair range (Figure S11, Supporting Information). However, when the CpG site was located 30 base pairs away from the pegRNA cleavage site, no significant improvement in editing efficiency was observed for epiPE2 compared to lentiPE2, with both efficiencies very low (below 0.5%). These findings suggest that the methylation‐induced enhancement of PE efficiency is most pronounced when CpG sites are within 19 to 30 base pairs of the cleavage site.

### 2.6 Efficient epiPE2 Mediated Edits of Different Types in Different Human Cells

**Figure 3:**
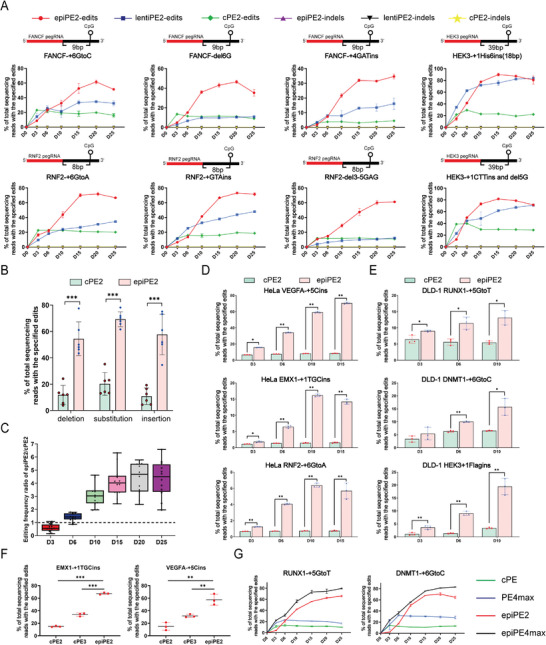
Efficient epiPE2 system in human cells. A) Comparison of cPE2, lentiPE2 and epiPE2 efficiencies at different time points in HEK293T cells. B) Statistical analysis of the deletion, substitution, and insertion efficiencies of cPE2 and epiPE2 in HEK293T cells. C) Overall editing frequencies induced by cPE2 and epiPE2. The cPE2 editing efficiency for each target was normalized to 1. D) Comparison of the efficiencies of cPE2 and epiPE2 at different time points in HeLa cells. E) Comparison of the efficiencies of cPE2 and epiPE2 at different time points in DLD‐1 cells. F) Estimated editing efficiency frequencies induced by cPE2, cPE3 and epiPE2. G) Comparison of the efficiencies of cPE2, cPE4max, epiPE4max and epiPE2 at different time points. Frequencies (means ± s.e.m.) were calculated from three independent experiments (n =  3). *P* values were obtained via two‐tailed Student's t tests. *P < 0.05, **P < 0.01 and ***P < 0.001.

To investigate the editing efficiency of the epiPE2 system at other genomic loci in human cells, we designed 18 types of edits, including small deletions, small insertions, and substitutions, targeting seven genetic loci in HEK293T cells (**Figure** **3**A; Figure S12, Supporting Information). From these designs, eight pegRNAs were selected to compare the efficiencies of the epiPE2, cPE2 and lentiPE2 systems.

The results showed that the epiPE2 efficiency outperformed that of cPE2 at all eight loci and that of lentiPE2 at six of the eight loci tested. Comparing to cPE2, the epiPE2 system demonstrated significant improvements in desired edits, ranging from 2.0‐ to 38.2‐fold increases in all tested pegRNAs. DNMT1 (+1ATCins) editing resulted in the greatest improvement (38.2‐fold increase) on Day 25. Notably, the relative improvement in epiPE2 over cPE2 appeared to be time dependent. The highest efficiency of cPE2 observed between Day 3 and Day 6, followed by a slight decrease. Conversely, epiPE2 demonstrated a robust increase in editing efficiency from Day 3, peaking on Day 15 or Day 20, with the maximal editing efficiency reaching 89.8% (HEK3 +1His6ins, Day 15) (Figure 3A). Across different editing types, epiPE2 presented average increases of 4.8×, 3.4×, and 5.3× for small deletions, point mutations, and small insertion edits, respectively (Figure 3B). On average, compared with cPE2, epiPE2 achieved 3.4×, 4.6×, 5.3×, and 5.4× increases in all edits on Days 10, 15, 20, and 25, respectively (Figure 3C).

Interestingly, in the two editing types with similar efficiencies between lentiPE2 and epiPE2, the closest CpG sequence was located 39 bp (i.e., >30) downstream of the predicted cleavage site of the pegRNA, which is consistent with the earlier finding that when the CpG site is greater than 30 bp away, the improvement by epiPE2 is minimal.

We then conducted experiments to validate the performance of the epiPE2 system in HeLa and DLD‐1 cells. Consistent with the results obtained in HEK293T cells, the editing efficiencies of epiPE2 was significantly greater than those of cPE2, with average increases of 8.7‐fold in HeLa cells and 2.9‐fold in DLD‐1 cells (Figure 3D,E).

We also tested two different primary fibroblast cell types: human fetal lung fibroblasts (HFL1), which have limited in vitro passaging capacity (2‐3 passages), and porcine fetal fibroblasts (PFF), which can proliferate for up to 2 months in culture. We compared the gene editing efficiency between the epiPE2 and cPE2 systems in these two cell lines. Notably, epiPE2 significantly enhanced editing efficiency in both cell lines, with a more pronounced effect observed in PFF cells, with and editing efficiency increase by 10 to 32 folds (Figure S13A,B, Supporting Information).

#### 2.6.1 Low Levels of Off‐Target Editing by epiPE2

We assessed the off‐target effects of both cPE2 and epiPE2 systems (Figure S14, Supporting Information). We evaluated sgRNA‐independent off‐target effects by editing the VEGFA gene in HEK293T cells over 10 days using both systems. Genomic DNA was extracted from wild‐type, cPE2‐edited, and epiPE2‐edited cells, and whole‐genome sequencing was performed. No significant differences in base mutations or indels were detected, indicating that both systems exhibit high specificity and minimal off‐target effects (Figure S15, Supporting Information).

### 2.7 Minimal Oncogenic Effects of epiPE2 Edited Cells

To investigate potential oncogenic effects of prolonged gene editing with EBNA‐1, we analyzed gene expression and cell proliferation in mCherry‐reporter cells after epiPE2‐mediated editing. A stable mCherry‐reporter cell line was transfected with epiPE2 targeting the mCherry gene. After 10 days, RNA was extracted from both transfected and untransfected cells, and transcriptome sequencing was performed. Genes with a p‐value < 0.01 and a fold change > 2 or < −2 were considered significantly altered. Of the 63140 genes analyzed, only 91 (0.15%) showed differential expression. Pathways related to actin filament bundle assembly and immune response activation were most affected, but no cancer‐related genes were identified (Figure S16A,B, Supporting Information).

#### 2.7.1 epiPE2 Does Not Affect Cell Proliferation

To assess the impact of epiPE2 on cell proliferation, we performed a CCK8 assay on epiPE2‐transfected HEK293T cells. After 15 days of epiPE2‐transfected, we compared the proliferation of these cells with unedited HEK293T cells. The results indicated no significant differences in proliferation between the two groups over the 3‐day observation period. Consequently, we conclude that epiPE2 does not affect the proliferative capacity of HEK293T cells (Figure S17, Supporting Information).

### 2.8 Minimal Off‐Target DNA Methylation by epiPE2

We also assessed DNA methylation at nontargeted sites in edited cells. No significant methylation differences were observed between control and epiPE2‐edited cells (Figure S18A, Supporting Information). To further examine potential effects on genome‐wide DNA methylation, we performed whole‐genome methylation sequencing in three groups: wild‐type, cPE2‐transfected, and epiPE2‐transfected cells (n = 3 per group). No significant variations in methylation levels or differentially methylated regions (DMRs) were found (Figure S18B,C, Supporting Information). Additionally, no off‐target methylation was observed at sequence‐dependent off‐target sites (Figure S18D, Supporting Information). These results suggest that, despite increased expression of methyltransferases, the likelihood of off‐target methylation events is low. The above results suggest that epiPE2 is a relatively safe editing tool.

### 2.9 The epiPE Strategy is Compatible with Other PE Systems

Although current work of epiPE is PE2 based, our strategy is not limited to PE2. Rather, by its design, it's compatible with all other PE systems. To demonstrate this, we first compared the efficiencies by epiPE2 with a newer version of PE system, namely PE3. epiPE2 outperformed cPE3 in efficiency at three loci in HEK293T cells (Figure 3F; Figure S19, Supporting Information). After including EBNA1/oriP to PE3 to construct epiPE3, we performed edits at the EMX1, HEK3, VEGFA, and RNF2 loci to compare the outcome by epiPE3 and lentiPE3. Our results show that epiPE3 exhibited significantly higher editing efficiency than lentiPE3 across all time points (Figure S20, Supporting Information).

PE4max is among the latest version of PE, which was reported to increase PE2 efficiency by an average of 7.7‐fold, via several pegRNAs (Figures 1E,G,I and 3G; Figure S21, Supporting Information). Here we constructed epiPE4max, and compared it with cPE2, epiPE2, and cPE4max. Unexpectedly, the results demonstrated that epiPE2 is the best performer with the highest efficiencies at different loci: RUNX1 +5 G to T, DNMT1 +6 G to C, and HEK3+1Flagins. Notably, both epiPE4max and cPE4max exhibited lower editing efficiencies for longer insertions (e.g, HEK3+1Flagins, insert 24 bp, Figure S21, Supporting Information) when compared to cPE2; whereas for the same edit, epiPE2 outperformed cPE2. This finding aligns with previous studies,^[^ ^1^ ^]^ suggesting that the MLH1dn domain may lose its ability to enhance editing efficiency, thus explaining the superior performance of epiPE2 over epiPE4max. These results indicate that epiPE2 is a more efficient prime editing tool in human cells.

### 2.10 One‐Step Generation of Multiple Precise Genome Editing Events by Csy4‐Mediated epiPE2 System

**Figure 4:**
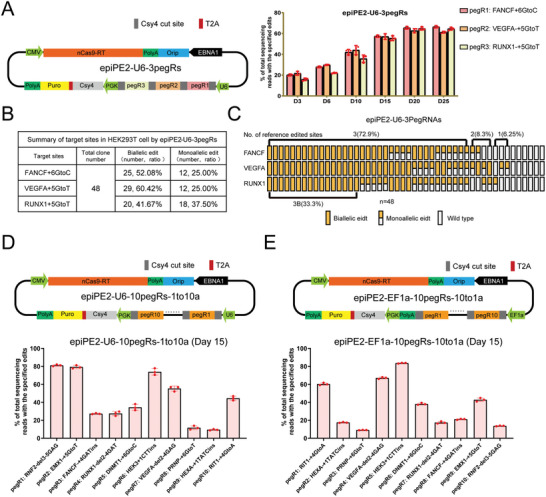
One‐step generation of multiple precise genome edits via the Csy4‐mediated processing system. A) Structure of the vectors of three‐pegRNAs containing cPE2 and epiPE2 and statistical analysis of the efficiencies of individual pegRNAs. B) Summary of mutation frequencies at three target sites by the three‐pegRNA epiPE2‐Csy4 system in HEK293T cells. C) Schematic representation of the genotyping results for all 48 cell colonies categorized as biallelic mutation, monoallelic mutation and wild type. D) Structure of the vector used to express pegRNAs 1 to 10 via the U6 promoter and efficiency analysis. E) Structure of the vector used to express pegRNAs 1 to 10 via the EF1α promoter and efficiency analysis.

After establishing that epiPE2 significantly enhances prime editing efficiency while maintaining a low indel safety profile, we utilized the system for multiplex prime editing, which has rarely been explored in previous studies. To achieve this, we incorporated a PGK‐Csy4 cassette into the epiPE2 vector, which we refer to as epiPE2‐Csy4. Csy4, which is responsible for CRISPR transcript processing in *Pseudomonas aeruginosa*,^[^ ^23^ ^]^ was hypothesized to assist in improving the design and expression of multiple individual pegRNAs (**Figure** **4**A, left panel). We constructed a three‐pegRNA sequence in tandem flanked by Csy4‐targeted sites in the epiPE2‐Csy4 vector and transfected it into HEK293T cells. We used high‐throughput sequencing to evaluate the efficiencies of the three pegRNAs from Day 3 to Day 25 posttransfection (Figure 4A, right panel). All three pegRNAs achieved efficiencies of approximately 60%. We then selected 48 single‐cell colonies and conducted genotyping via PCR‐Sanger sequencing. The results revealed that pegRNA1 (FANCF+6GtoC) had 52.08% efficiency (25/48) in biallelic editing and 25% (12/48) efficiency in monoallelic editing; pegRNA2 (VEGFA+5GtoT) had 60.42% efficiency (29/48) in biallelic editing and 25% (12/48) efficiency in monoallelic editing; and pegRNA3 (RUNX1+5GtoT) had 41.67% efficiency (20/48) in biallelic editing and 37.5% (18/48) efficiency in monoallelic editing (Figure 4B). Notably, 72% (35/48) of the colonies contained edits in all three genes, and nearly half (16/35) had biallelic edits in all three genes (Figure 4C). Interestingly, no unwanted genotypes were observed in the cloned populations. We hypothesize that this outcome is primarily due to the epiPE2 editing process, which predominantly induces single‐strand DNA breaks, leading to a low frequency of indels. Consequently, the genotypes within the clones remain highly uniform. These results demonstrate that epiPE2‐Csy4 is an efficient multiplex prime editing system in human cells.

We subsequently aimed to determine the upper limit of multiplex targeting via the epiPE2‐Csy4 system. To achieve this goal, we selected ten pegRNAs, assembled them into the epiPE2‐Csy4 vector, and transfected them into HEK293T cells (Figure 4D). The mutation frequencies were determined 15 days posttransfection, and we observed various efficiencies for each pegRNA, with the highest efficiency of up to 81.26% observed for pegRNA 1 (RNF2‐del3‐5GAG) (Figure 4D). Interestingly, we found that U6, a polIII‐dependent promoter typically used to initiate the transcription of small RNAs^33^, drove the expression of the pegRNA array, which was over 1 kb in size. We further explored whether the use of the polII‐dependent EF1α promoter could improve the expression of the pegRNAs in the array (Figure 4E). Our results showed that the EF1α promoter also achieved successful multiplex editing, with the highest efficiency observed for pegRNA (HEK3+1CTTins), at 83.5%. We also monitored the promoter effects of the pegRNAs between the U6‐, EF1α‐, and CBh‐promoter driving vectors. The results revealed that the U6 promoter‐driving vector had the greatest efficiencies for the first five pegRNAs, and the polII‐dependent promoter outperformed the U6 promoter for pegRNAs 6 to 10 (Figure S22, Supporting Information). Indel rates were comparable on Day 15 posttransfection (Figure S23, Supporting Information). Overall, our findings suggest that the epiPE2‐Csy4 system is effective for multiplex targeting, with the U6 promoter suitable for up to five pegRNAs. This is likely due to the U6 promoter's reduced efficiency in initiating long transcripts. The total length of the ten pegRNAs is approximately 1.4 kb, with the first five pegRNAs totaling around 0.7 kb. Within this range, the U6 promoter demonstrates better synthesis capability. However, for arrays containing more pegRNAs, promoter optimization may be necessary.

## 3 Discussion

Prime editors are capable of generating versatile edits, including all types of base conversions, small‐sized insertions, and deletions.^[^ ^6^, ^7^, ^8^ ^]^ Recent data have demonstrated that PEs can also achieve large gene insertions or deletions.^[^ ^1^, ^24^, ^25^ ^]^ However, the underlying repair mechanism of PEs remains unclear, limiting the development of more efficient gene editing tools. In this study, we demonstrate that alterations in methylation marks at the target region are essential for PE2 editing. Furthermore, extending the editing duration can further improve editing efficiency. Based on this, we developed epiPE2 by incorporating the episomal element EBNA1/oriP into the prime editing system. We show that, compared to PE2, PE3, and PE4max, epiPE2 significantly enhances editing efficiency in human cells across all tested loci, resulting in a 2.0‐ to 38.2‐fold increase in efficiency, while maintaining low indel rates.

We attribute the improvement achieved by epiPE2 to two mechanisms: (i) modulation of the methylation marks as a means to avoid MMR at the target locus and (ii) prolonged expression of the PE components. With respect to the first mechanism, MMR is a highly conserved process during DNA replication and plays an important role in safeguarding genome integrity. In mammals, during DNA replication or repair processes, strand discrimination is achieved by recognizing the nick‐containing strand as the nascent strand,^[^ ^26^, ^27^, ^28^ ^]^ and this process is harnessed in the development of the base editor and PE3/PE3b and PE5 systems.^[^ ^2^, ^7^, ^8^, ^29^ ^]^ Several works have suggested that the difference in methylation levels between the parent (generally higher methylation level) and daughter strands (generally no or low methylation level) may also guide strand discrimination.^[^ ^13^, ^14^, ^30^ ^]^ Intriguingly, our data suggest that the prime editing process (without EBNA1/oriP) downregulates the overall methylation level around the target region (e.g., lentiPE2 vs WT cells, Figure 1F). This is probably because the prime edited/newly synthesized strand has low methylation. In contrast, in epiPE2‐treated cells, overall methylation levels are high at the target locus. This observation suggests that EBNA1/oriP promotes methylation in the newly synthesized/edited strand, making them similar to/indistinguishable from the unedited strand in this respect, leading to escape from MMR and consequently higher PE rates. This finding is in line with early reports that EBNA1/oriP promotes methylation in mammalian cells.^[^ ^16^, ^17^ ^]^

To further clarify the mechanisms of *de novo* DNA methyltransferase in PE editing, we performed qPCR to compare the mRNA expression levels of the DNMT3A and DNMT3B genes in cells transfected with cPE2 and epiPE2, and the results revealed that the mRNA expression levels of DNMT3A and DNMT3B were significantly increased. Previous studies have established a significant connection between DNA methyltransferases (DNMTs) and DNA damage and repair.^[^ ^11^, ^30^ ^]^ However, this study revealed for the first time that the expression levels of DNMT3A and DNMT3B can be upregulated during the PE editing process. This finding strongly implies their potential involvement in the repair pathway of PE. To further validate their function in the PE editing process, we generated HEK293T cells with DNMT3A and DNMT3B knockout and overexpression, respectively. The results revealed that the editing efficiencies of cPE2 and epiPE2 were significantly lower in DNMT3A‐ and DNMT3B‐knockout cells but significantly greater in DNMT3A‐overexpressing cells. Moreover, it is worth noting that some loci had decreased efficiency in the DNMT3B‐knockout cell line and increased efficiency in the DNMT3B‐overexpressing cell line, whereas other loci had decreased efficiency in both types of cell lines. These results indicate that DNMT3B may play a more complex role in the process of PE editing, a possibility that warrants further investigation (Figure 2).

By using Csy4‐mediated processing of the pegRNA array, we demonstrated that PE can efficiently edit up to 10 loci in human cells, likely as a result of both the high editing rate and the prolonged editing time window provided by epiPE2. To address this issue, we explored the use of multiple U6 promoters to drive the expression of pegRNAs. We used three U6 promoters to trigger the expression of three different pegRNAs individually and assessed the editing efficiencies over a period of 25 days. Encouragingly, the results demonstrated that the epiPE2 system effectively achieved high editing efficiency of multigene editing mediated by these independent U6 promoters (Figure S12, Supporting Information). Yuan and colleagues reported PE‐mediated multiplex editing at 3 loci via drive‒and‒process (DAP) CRISPR array architectures,^[^ ^31^ ^]^ which represents an alternative strategy to achieve multiplexed PE editing. The efficiency of the PE system in multi‐gene editing is closely tied to its exceptionally low indel occurrence rate, which ensures a minimal error rate in edited cells. Our results demonstrate that while epiPE2 significantly enhances the editing efficiency of PE2, it does not increase the frequency of indels. This is primarily because the PE2 system only cleaves one DNA strand, rather than inducing double‐strand breaks. We believe that all these efforts will find applications in treating complex genomics and polygenic diseases.

Although we have demonstrated that methylation of the edited strand plays a crucial role in enhancing the editing efficiency of PE, several mechanisms still require further clarification in future studies. For instance, it remains unclear to what extent the newly synthesized edited strand is methylated prior to each round of MMR repair, as well as the precise magnitude of the enhancement driven by epiPE2. Although current technical limitations make it difficult to directly address these questions, we aim to further investigate this in future work. Additionally, while we have conducted extensive experiments showing that epiPE improves editing efficiency by promoting repair of the edited strand, and have preliminarily linked this process to the MMR repair pathway, additional experiments are necessary to confirm the direct relationship between the two. Furthermore, questions such as how oriP/EBNA1 increases methylation in a target‐specific manner but not genome‐wide, and how increased methylation does not silence the episomal plasmid as it typically does in other contexts, also warrant further exploration.

## 4 Experimental Section

### Plasmids Generation

pegRNA‐expressing cassette and a puromycin‐expressing sequence obtained by amplification from pU6‐pegRNA‐GG‐acceptor (Addgene:132 777) and pKLV2‐U6gRNA5(BbsI)‐PGKpuro2AZsG‐W (Addgene:67 975), respectively. These components were then integrated into the pCMV‐PE2 plasmid (Addgene:132 775) and pCMV‐PEmax‐P2A‐hMLH1dn (Addgene:174 828) to construct the cPE2 and cPE4max vectors, respectively. The epiPE2 plasmid was created by amplifying the EBNA1‐oriP fragment from pCEP4 (Thermo) and subsequently combining it with cPE2. To produce the epiPE2‐Csy4 vector, the Csy4 expression cassette was synthesized by blocking the AarI site (Genscript) and fused to epiPE2. The pLenti‐PE2‐BSD vector was purchased from Addgene (Addgene:161 514).

PCR amplifications were carried out using the KAPA HiFi PCR Kit (Roche), while vectors were predominantly linearized using restriction digestions. Gel extraction of the amplified PCR fragments was performed using the FastPure Gel DNA Extraction Mini Kit (Vazyme). The ClonExpress II One Step Cloning Kit (Vazyme) was used to construct cPE2, cPE4max, epiPE2, and epiPE2‐Csy4 vectors. PegRNAs were ordered from Genscript and introduced to epiPE2 or epiPE2‐Csy4 (digested with AarI). The primers and pegRNA sequences utilized in this study were synthesized at Tsingke Biotechnology and were listed in Table S1‐6 (Supporting Information).

Plasmids were isolated using the Plasmid Mini Kit (Omega) and eluted in the kit provided elution buffer. All constructs were thoroughly validated by Sanger sequencing across all assembly junctions, and their coding sequences were fully confirmed.

### Cell Culture

HEK293T cells (ATCC), HeLa cells, HFL1 cells, PFF cells, K562 cells and DLD‐1 cells were utilized in this study. These cells were cultured in accordance with established protocols. Specifically, cells were maintained in Dulbecco's modified Eagle's medium (DMEM) containing GlutaMAX (Gibco), supplemented with 10% fetal bovine serum (FBS) and 1% penicillin‐streptomycin (Gibco) for HEK293T and HeLa cells, and in Roswell Park Memorial Institute (RPMI) 1640 medium plus GlutaMAX (Gibco) for DLD‐1 and K562 cells. PFF cells were maintained in DMEM medium supplemented with 15% fetal bovine serum (FBS), 1% GlutaMAX™ (Gibco, USA), 1% non‐essential amino acids (NEAA; Gibco), and 1% penicillin‐streptomycin (Gibco), with additional supplementation of 2.5 ng/mL basic fibroblast growth factor (bFGF). HFL1 cells were cultured in Ham's F‐12K medium containing 10% FBS and 1% penicillin‐streptomycin. All cells were cultured at 37 °C in incubators containing 5% CO_2_. Passage was carried out upon reaching 80% confluency. To ensure the absence of mycoplasma contamination in the cell culture medium, regular testing was conducted using the Mycoplasma Detector (Vazyme). The test was performed on a monthly basis.

### Transfection

HEK293T cells were passaged every two days at a split ratio of 1:5. For plasmid transfection, HEK293T cells were plated in 6‐well plates one day before transfection, and were transfected when the cells reaching 70%‐90% confluency. For each well on the plate, a total of 2 µg plasmid was used according to the operating instructions. Briefly, the transfection plasmid was added into 100 µL transfection buffer with 4 µL JetPRIME and incubated for 10 min at room temperature before pipette onto the supernatant. For PE3, 1500 ng cPE2 plasmids plus 500 ng nick sgRNA plasmid were used per well.

HeLa cells, K562 cells, HFL1 cells, PFF cells and DLD‐1 cells were cultured in T75 flasks and transfected using an electroporation instrument (Celetrix).^[^ ^31^ ^]^ Typically, for each well, a total 1×10^6^ cells were transfected with 2 µg plasmid using 20 µL electroporation cuvette where the cells were shocked with 500 V. After electrotransfection, cells were transfered into 6‐well plates supplied with 2 mL culture medium without penicillin‐streptomycin. 2 µg mL^−1^ puromycin (Gibco) was added to the culture medium 24 hours after transfection for the cPE2, cPE3, and cPE4max plasmid‐treated cells, and was withdrawn 72 hours post‐transfection. For the epiPE2 plasmid‐treated cells, puromycin was added 24 hours post‐transfection and maintained throughout the culture period. For cells infected with lentivirus PE, puromycin was added to the culture medium 72 hours post‐infection and maintained for the duration of the experiment.

### Cell Colonies and Genotyping

To generate single cell colonies, HEK293T cells treated with epiPE2‐U6‐3pegR and epiPE2‐EF1α‐3pegR were cultured for ten days post‐transfection. The cells were subsequently serially diluted to appropriate concentrations and seeded onto 100‐mm culture dishes containing 2 µg mL^−1^ puromycin. After twenty days of transfection, colonies of cells were observed and picked up using cloning cylinders (Corning). The lysates of the picked colonies were obtained by lysing the cells in NP‐40 solution (Beyotime) at 65 °C for 60 min, followed by incubation at 95 °C for 10 min. The lysis solution was utilized for PCR amplification, and primers were designed to amplify sequences containing the desired edits. Cell colonies genotyping was accomplished through PCR amplicon Sanger sequencing (Tsingke). To determine the editing status of each clone, Sanger sequencing was performed and chromatograms were analyzed. Clones with only wild‐type peaks were classified as wild‐type, while those with only edited peaks were classified as edited. Clones showing both wild‐type and edited peaks were classified as heterozygous, regardless of the peak ratio.

### Western Blot

To assess the expression of the PE component, cPE2, epiPE2 and lentiPE2 treated cells were harvested and subjected to lysis in RIPA buffer (Sigma). The total protein content of each sample was determined using a BCA kit (Beyotime). Equivalent amounts of total protein from each sample were separated via 12% sodium dodecyl‐sulphate polyacrylamide gel electrophoresis (SDS‐PAGE) and transferred onto a polyvinylidene difluoride membrane (Millipore). Following transfer, the membranes were blocked with 5% non‐fat milk and then probed with Cas9 and β‐actin antibodies at a 1:1000 dilution (Proteintech). Subsequently, the membranes were washed with TBST and incubated with HRP‐conjugated anti‐Rabbit IgG as the secondary antibody. The digital signal for chemiluminescent Western blotting was obtained using the Bio‐Rad GelDoc XR and ChemiDoc XRS system, and analysis was performed with the Quantity One program (Bio‐Rad).

### Quantitative RT‐PCR (qPCR) and Statistical Analysis

To investigate the mRNA expression level of DNMT3A, DNMT3B and PE, total RNA obtained from the samples was converted into cDNA using the NovoScript One‐Step RT‐PCR Kit (novoprotein). The qPCR was performed using NovoStart SYBR qPCR SuperMix Plus (novoprotein) on the CFX384 Touth Real‐Time PCR Detection System following the manufacturer's instructions (Bio‐Rad). The specific primer sequences used for qPCR were shown in Table S7 (Supporting Information). The qPCR was performed as follows: 1 cycle at 95 °C for 2 min, 40 cycles at 95 °C for 30 s, 60 °C for 20 s and 72 °C for 15 s. β‐actin (ACTB) was used as a control gene to normalize the expression levels. The student's t‐test was used to analyze the differential expression of genes.

### Off‐Target Analysis

Prediction of potential off‐target sites (OTS) was performed using the CRISPR‐offinder^48^ online software. The top four sites with the highest similarity to the pegRNA sequences were identified as potential OTS. To confirm the presence of any mutations at these sites, specific primers were designed for OTS amplification from cells treated with cPE2 and epiPE2 on day 20 post‐transfection. High‐throughput sequencing was performed to determine the occurrence of any mutations at these targeted sites. All sequences including off‐target sites and amplification primers were shown in Tables S8‐S9 (Supporting Information)

### Sample Preparation and High‐Throughput Sequencing

HEK293T, HeLa, and DLD‐1 cells were obtained on different days post transfection, and the DNA was extracted from them for analysis. Specifically, HEK293T cells were collected on days 0, 3, 6, 10, 15, 20, and 25, HeLa cells were collected on days 0, 3, 6, 10, and 15, K562 cells were collected on day 5, PFF cells were collected on days 5 and 10, HFL1 cells were collected on days 5 and 15, while DLD‐1 cells were collected on days 0, 3, 6, and 10. All the cells were lysed using freshly prepared lysis buffer (Beyotime) and incubated at 65 °C for 60 min, followed by incubation at 95 °C for 10 min. The resulting lysis solution was then subjected to PCR amplification of the target region using specific primers with barcodes. Equal amounts of PCR products were pooled, purified, and commercially sequenced (Annoroad) using the NovaSeq platform.

The obtained amplicon sequencing reads (FASTQ files) were then demultiplexed using bcl2fastq (Illumina) and analyzed by aligning the amplicon reads to a reference sequence by CRIPResso2^46^, utilizing standard mode (for point mutation) and HDR mode (for insertion and deletion edits). The prime editing efficiencies were calculated as the ratio of the number of desired reads to the total number of reads, while indel rates were calculated as the ratio of the number of indel‐containing reads to the total number of reads.

For the measurement of the methylation level of the target region, approximately 1 µg of genomic DNA was treated with sodium bisulfite using the DNA Bisulfite Conversion Kit (TIANGEN), following the manufacturer's instructions. The target region was then amplified using the Methylation‐specific PCR Kit (TIANGEN), and the resulting PCR products were pooled, purified, and commercially sequenced (Annoroad) using the NovaSeq platform. The obtained clean data reads were mapped to the reference sequences using the BWA‐MEM algorithm, and the methylation rates were calculated as the ratio of the number of methylated‐C‐containing reads to the total number of reads for the specific locus.

### Lentivirus Production and Cellular Infection

HEK293T cells were seeded onto 100 mm dishes in culture medium. When the cells reached approximately 90% confluency, they were transfected using JetPrime reagent. Briefly, a mixture of 12 µg lentiviral plasmid, 8 µg pMD2.G (Addgene: 12259), and 4 µg psPax2 (Addgene: 12260) was prepared in 1 mL transfection buffer, to which 100 µL JetPrime was added. The mixture was incubated for 10 minutes at room temperature, and then added to the cell culture medium. At 6 hours post‐transfection, the cells were refreshed with maintaining medium, and at 72 hours post‐transfection, the supernatant was collected and filtered through a 0.45 µm pore filter (Corning). The supernatant was then concentrated at 30000 × g using a high‐speed refrigerated centrifuge (Beckman) and stored at −80 °C.

For lentivirus transfection, HEK293T cells were seeded onto 6‐well dishes, and the concentrated lentivirus was added to the culture medium supplemented with 8 µg mL^−1^ Polybrene (Sigma). The cells were incubated overnight, and the culture medium was then replaced with normal culture medium. At 72 hours post‐transfection, puromycin was added to the culture medium and maintained throughout the culture period.

### Whole‐Genome Bisulfite Sequencing

Genomic DNAs (gDNA) were isolated using a DNA extraction kit (Takara, China) following the manufacturer's instructions. The isolated DNA content was measured using a NanoDrop ND‐2000 spectropho‐tometer (Thermo Scientific, USA). Take a 100–200 ng DNA sample, then add 1% λDNA, and disrupt by sonication. Suitable DNA fragments were selected and treated with Bisulfite to convert unmethylated “C” bases into “U” bases. The bisulfite‐converted DNA was fully denatured into single strands, an “A” base was introduced at the 3′ end, and an adaptor was attached at the 5′ end. PCR was used to selectively enrich DNA fragments with adaptor molecules ligated to both ends, and the DNA library amplified. Products were quantified using the Agilent high sensitivity DNA assay on a Bioanalyzer 2100 system (Agilent). Libraries were then sequenced on the Novaseq 6000 platform (Illumina) by Shanghai Personal Biotechnology Cp. Ltd.

### CCK8 Assay

Cells were seeded and cultured at a density of 4 × 10^3^/well in 100 µL of medium into 96‐well microplates. At different time points, 24 h, 48 h, and 72 h, cells were treated with 10 µL of CCK8 reagent per well and incubated for 1 hour. Cell viability was analyzed using the Cell Counting Kit‐8 (CCK8, Abbkine) according to the manufacturer's protocol. Each experiment was independently repeated three times. The absorbance at 450 nm was analyzed using a microplate reader (Agilent) to express the proliferation of cells.

### Statistical Analysis

The statistical analysis of the data was performed using GraphPad Prism 8 software. The analysis was based on three independent experimental replicates. To determine the statistical significance of the results, a two‐tailed Student's t‐test was employed. The indel rates were classified as being below the detection threshold (i.e., <0.2%), and therefore labeled as 0%. The data were presented as means ± standard deviation (SD).

## Conflict of Interest

The authors declare no conflict of interest.

## Author Contributions

X.H., X.X. (Xianghua Xu), Y.X., and G.Z. contributed equally to this work. S.Z., J.R., X.L., and X.H. designed the project. Most of the experimental work was co‐conducted by G.Z., Y.X., X.X. (Xianghua Xu), and X.H. with minor contributions from Y.S. and Y.L., R.H., S.L., X.X. (Xiaoning Xi), X.W., J.X., H.W., X.X., and Y.Z. analyzed the data. X.H., J.R., X.X. (Xuewen Xu), C.Z., and S.X. wrote the manuscript. X.L., J.R., and S.Z. supervised the project.

## Supporting information



Supporting Information

Supplementary Tables

## Data Availability

The deep‐sequencing data will be available on the NCBI Sequence Read Archive (SRA). The plasmid materials can be requested from corresponding authors.
