# Supplementary material for: Enhancing Prime Editing Efficiency Through Modulation of Methylation on the Newly Synthesized DNA Strand and Prolonged Expression
Source: Adv Sci (Weinh). 2025 Mar 7;12(17):2417790. doi: 10.1002/advs.202417790 (PMC12061241; doi:10.1002/advs.202417790)
Supplement: Supplementary file 1 — Supporting Information [file ADVS-12-2417790-s002.pdf]

ADVANCED SCIENCE
Open Access

Supporting Information

for *Adv. Sci.*, DOI 10.1002/advs.202417790

Enhancing Prime Editing Efficiency Through Modulation of Methylation on the Newly Synthesized DNA Strand and Prolonged Expression

Xiaosong Han, Xianghua Xu, Youcai Xiong, Guangxing Zhao, Ruigao He, Yinyu Su, Sheng Li, Changzhi Zhao, Xiaoning Xi, Yunxia Zhao, Xuewen Xu, Shengsong Xie, Heng Wang, Xinyun Li, Shuhong Zhao* and Jinxue Ruan**

Supplementary information

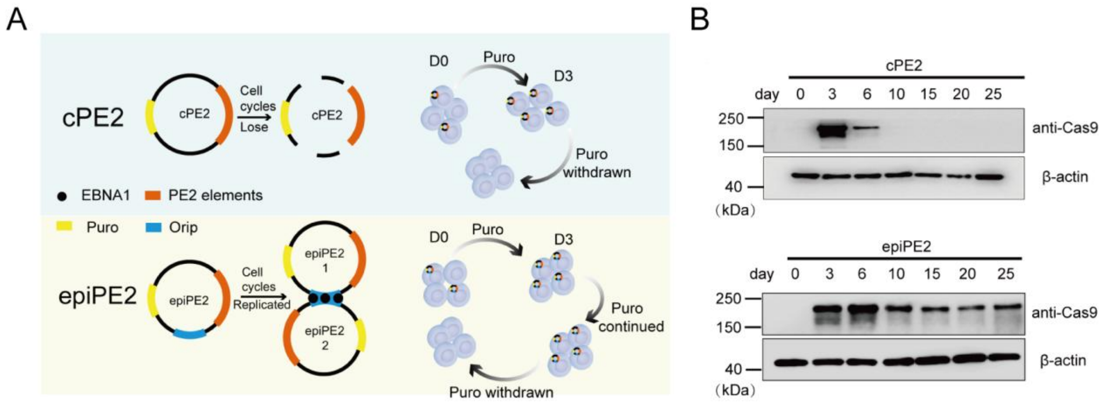

Supplementary Figure 1. Western blot analysis the protein expression between cPE2 and epiPE2 on different time points post transfection. A. Diagram of epiPE2 strategy. The cPE2 protein decreased with plasmid degradation gradually during cell cycles. The epiPE2 protein continuous express and enriched by drug selection, leading a higher efficiency. **B.** HEK293T cells were treated with epiPE2 and cPE2 plasmids according to the flowchart(Fig. 1a, right panel), the protein were collected on days 0, 3, 6, 10, 15, 20 and 25 post transfection. The positions indicated by the numbers on the left correspond to the marker bands for the respective protein sizes.

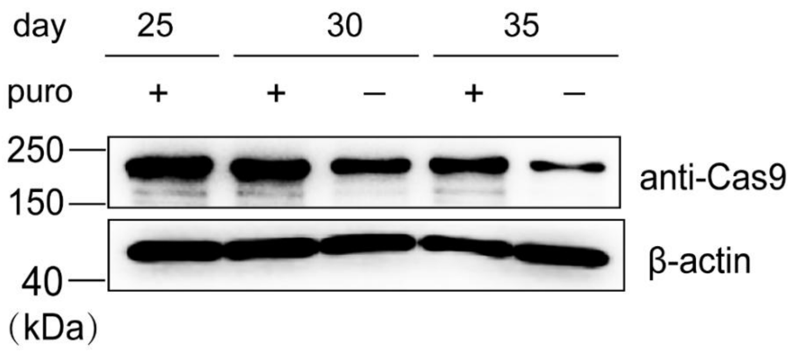

Supplementary Figure 2. Western blot analysis reveals changes in protein expression in epiPE2-treated cells upon withdrawal of drug selection. HEK293T cells were treated with epiPE2 plasmid according to the flowchart, and withdraw the puromycin on day 25 post transfection. The positions indicated by the numbers on the left correspond to the marker bands for the respective protein sizes.

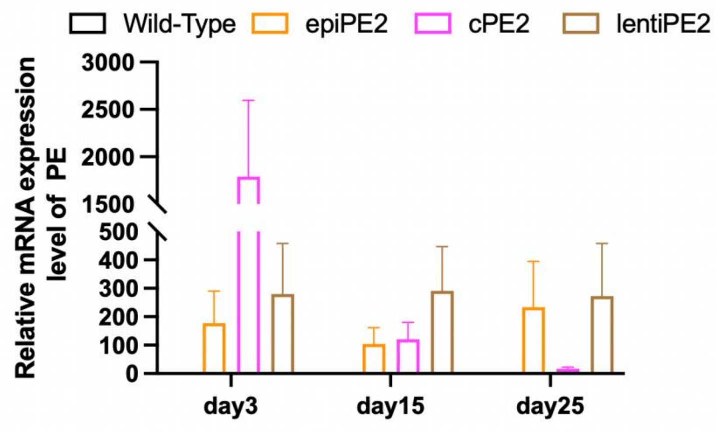

Supplementary Figure 3. Comparing the mRNA expression levels of PE in the lentiPE2 and epiPE2 editing systems.

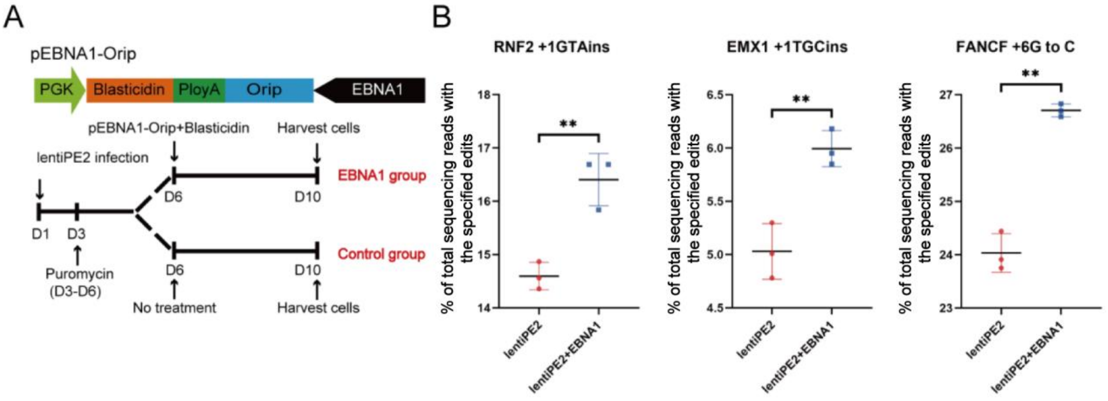

Supplementary Figure 4. EBNA1/oriP element promote the editing efficiency of lentiPE2 system. A. Experimental workflow for EBNA1-treated and control cells. **B.** Analysis of the editing efficiency in the lentiPE2 system with or without the EBNA1/oriP plasmid. EBNA1 group is represented by "lentiPE2+EBNA1", and Control group is represented by "lentiPE2".

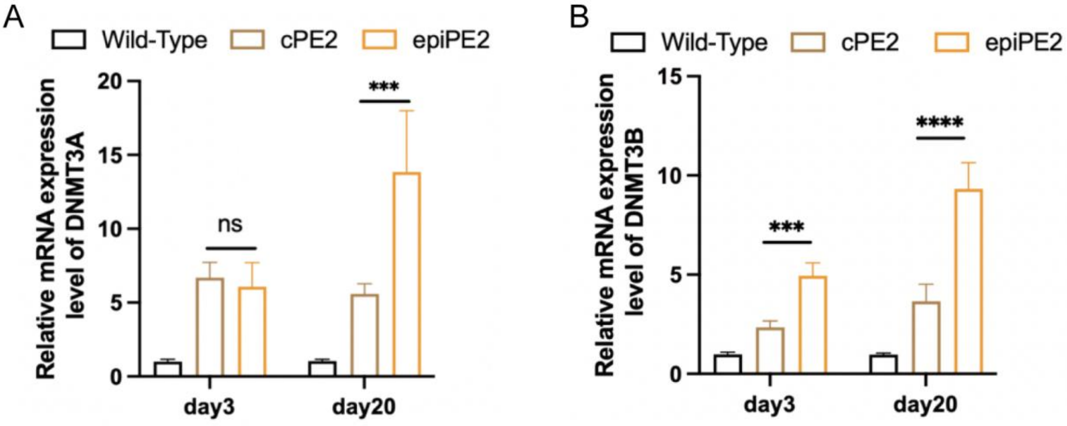

Supplementary Figure 5. Comparing mRNA expression levels of the DNMT3A and DNMT3B genes between cPE2 and epiPE2 at different time points.

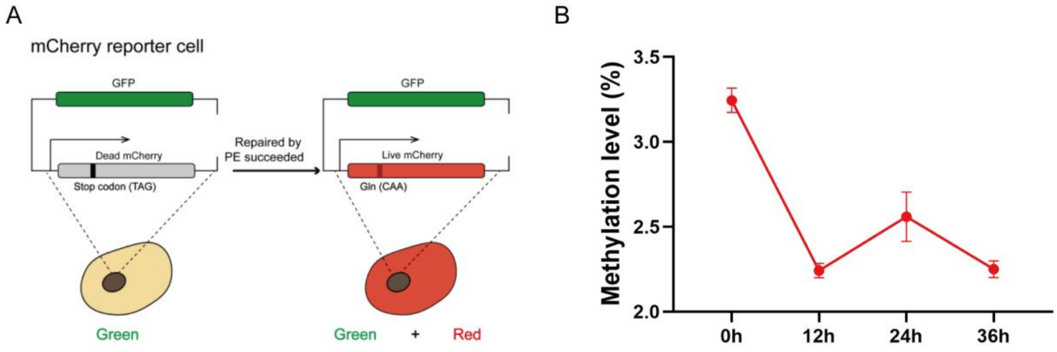

Supplementary Figure 6. Overview of mCherry reporter cell Line and early methylation changes during cPE2 editing. A. Schematic diagram of mCherry reporter cell line. B. Early Changes in Methylation Levels in mCherry Reporter Cells During cPE2 Editing.

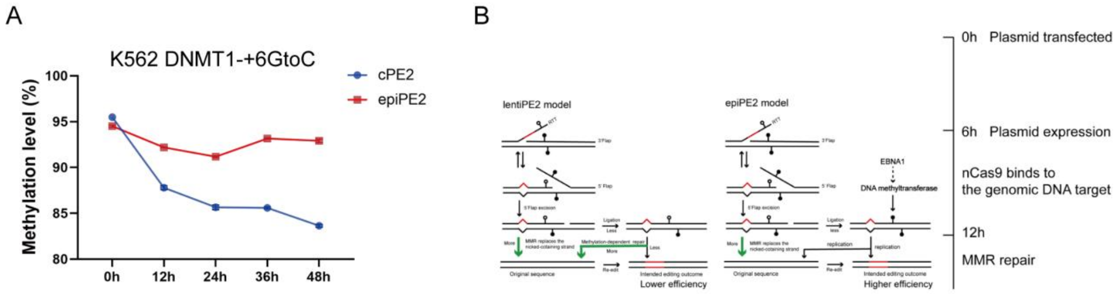

Supplementary Figure 7. Early methylation changes and pre-MMR events during PE editing in K562 cells. A. Early Changes in Methylation Levels in K562 Cells During PE Editing. B. Events that occurred at earlier points in time prior to MMR.

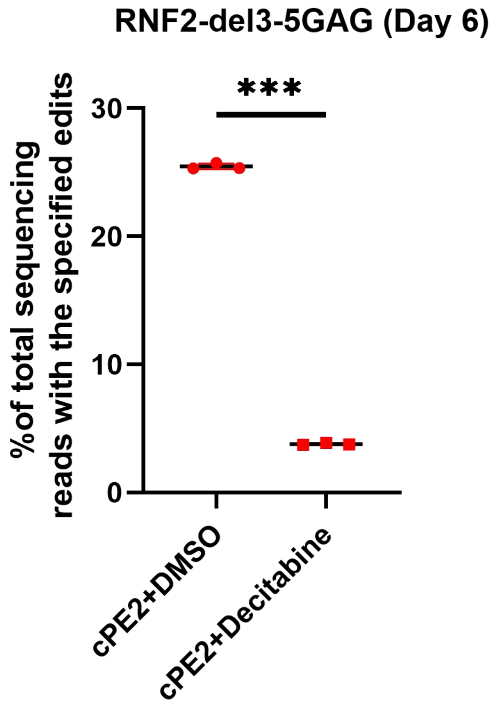

Supplementary Figure 8. Effect of small molecule inhibitors on PE editing efficiency at the RNF2-del3-5GAG site.

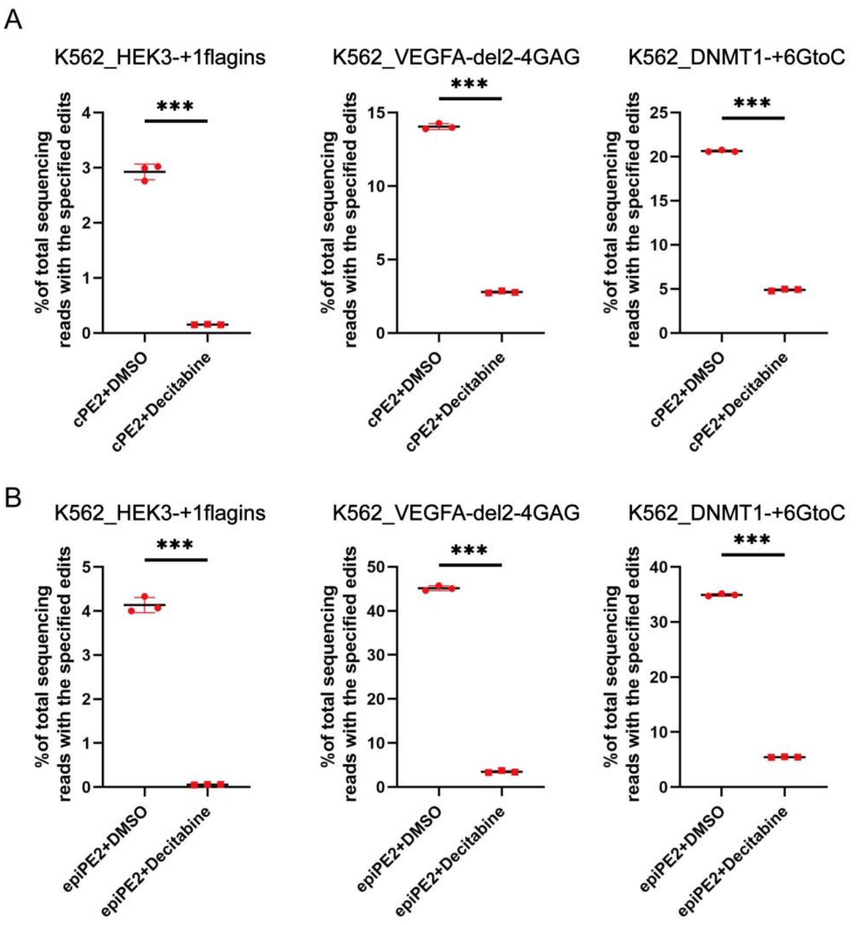

Supplementary Figure 9. Effect of small molecule inhibitors on PE editing efficiency in K562 cells. A. Effect of small molecule inhibitors on cPE2 editing efficiency in K562 cells. B. Effect of small molecule inhibitors on epiPE2 editing efficiency in K562 cells.

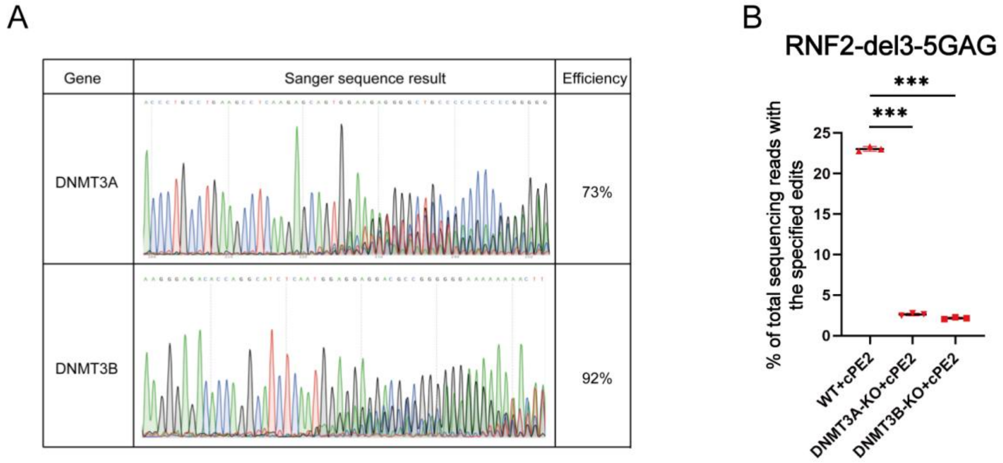

Supplementary Figure 10. Efficiency of DNMT3A and DNMT3B knockout in cell pools and their impact on PE editing at the RNF2-del3-5GAG Site in DNMT3A- and DNMT3B-knockout clones. A. Efficiency test for DNMT3A and DNMT3B knockout cell pools at day 5. B. Effect of DNMT3A and DNMT3B knockout on PE editing efficiency at the RNF2-del3-5GAG site.

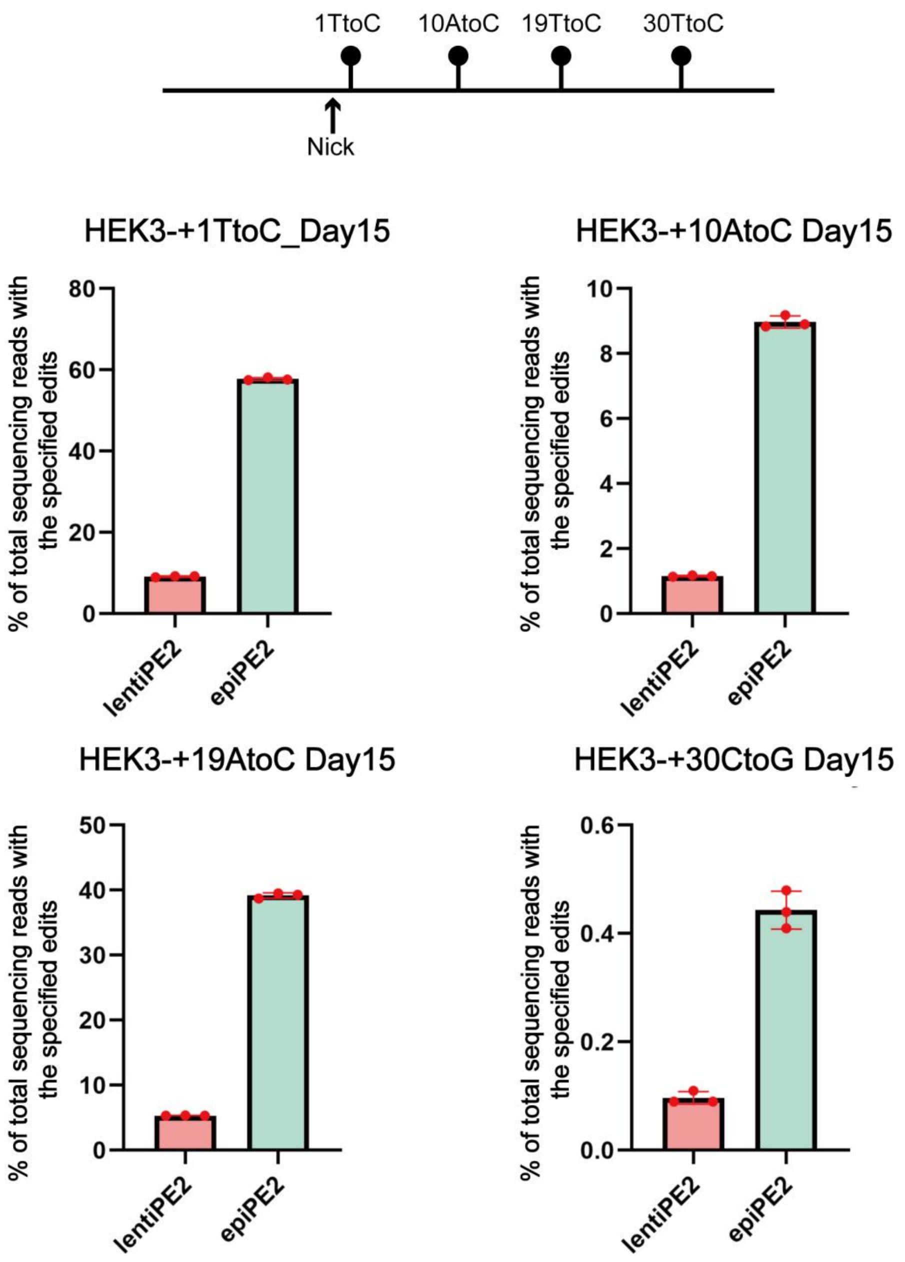

Supplementary Figure 11. Impact of introducing CpG sites at different distances on PE editing efficiency

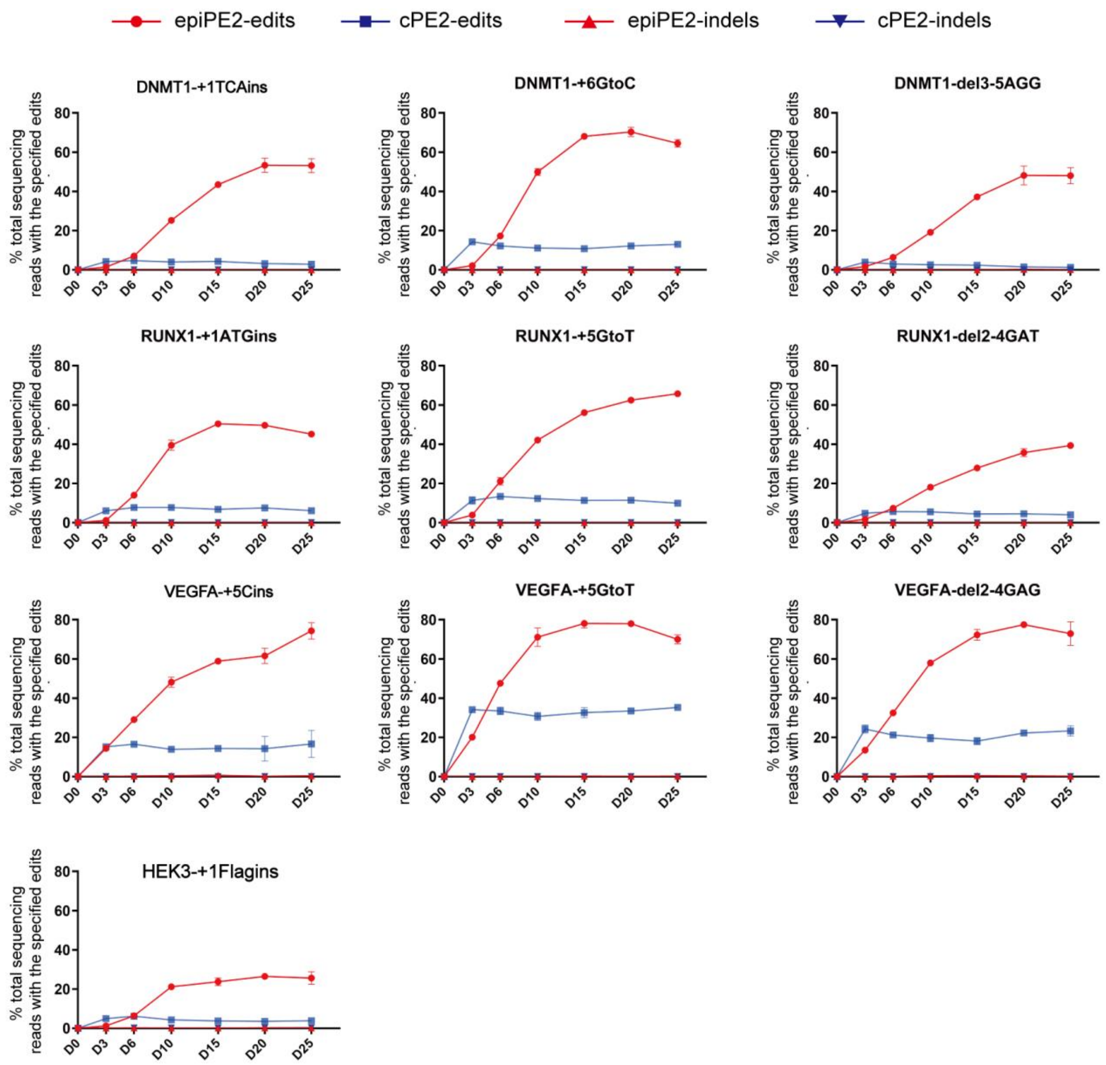

Supplementary Figure 12. Comparison of the efficiency between cPE2 and epiPE2 in different time points in HEK293T cells.

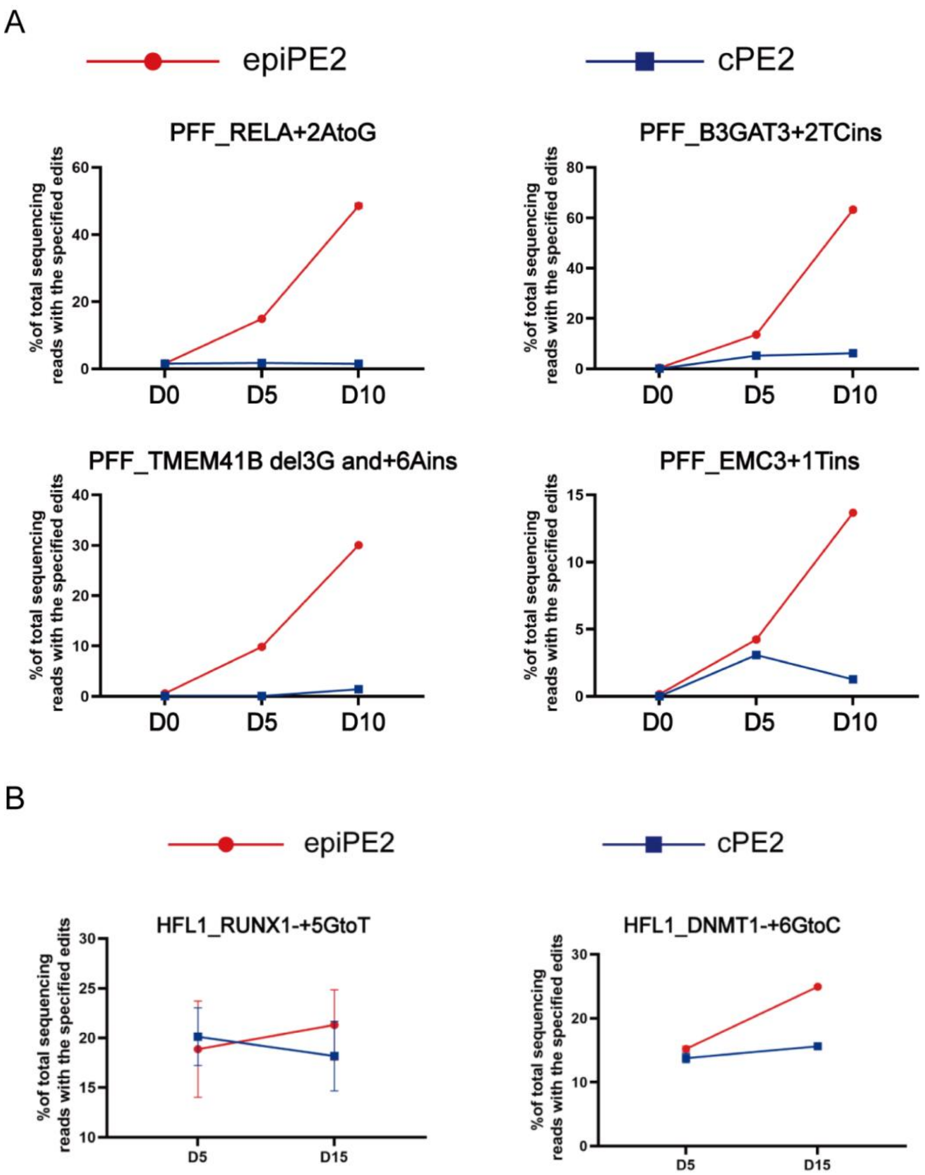

Supplementary Figure 13. Comparison of editing efficiencies between cPE2 and epiPE2 in PFF and HFL1 cells. A. Comparison of Editing Efficiencies between cPE2 and epiPE2 in PFF Cells. B. Comparison of Editing Efficiencies between cPE2 and epiPE2 in HFL1 Cells.

A

epiPE2-mediated on-target and off-target editing % (up) and indel % (below)			
pegRNA	RNF2-del3-5GAG	EMX1-+1TGCins	VEGFA-+5Cins
on-target	**59.98**	**61.01**	**68**
	<0.2	<0.2	<0.2
off-target 1	<0.2	<0.2	<0.2
	<0.2	<0.2	<0.2
off-target 2	<0.2	<0.2	<0.2
	<0.2	<0.2	<0.2
off-target 3	<0.2	<0.2	<0.2
	<0.2	<0.2	<0.2
off-target 4	<0.2	<0.2	<0.2
	<0.2	<0.2	<0.2

B

cPE2-mediated on-target and off-target editing % (up) and indel % (below)			
pegRNA	RNF2-del3-5GAG	EMX1-+1TGCins	VEGFA-+5Cins
on-target	**12.45**	**11.42**	**9.08**
	<0.2	<0.2	<0.2
off-target 1	<0.2	<0.2	<0.2
	<0.2	<0.2	<0.2
off-target 2	<0.2	<0.2	<0.2
	<0.2	<0.2	<0.2
off-target 3	<0.2	<0.2	<0.2
	<0.2	<0.2	<0.2
off-target 4	<0.2	<0.2	<0.2
	<0.2	<0.2	<0.2

Supplementary Figure 14. Off-target analysis and comparison between cPE2 and epiPE2 systems. Potential off-target sites were predicted by CRISPR-offinder software and subsequently amplified by specific primers measured by high-throughput sequencing.The data was analyzed by CRISPResso2.

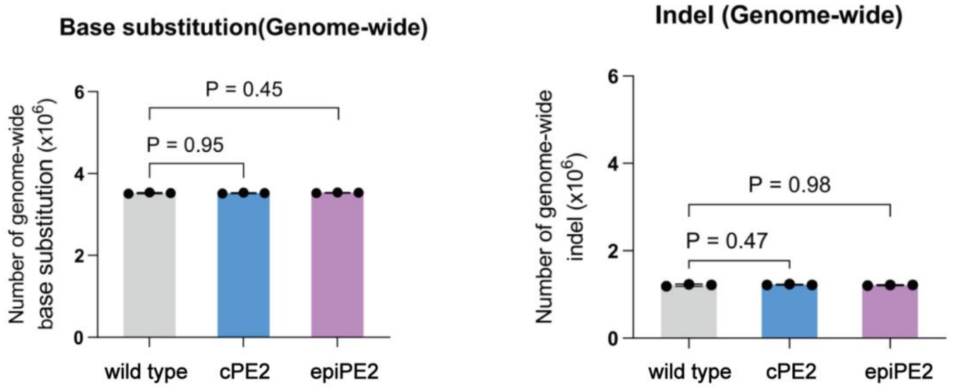

Supplementary Figure 15. Impact of PE editing on sgRNA-independent base substitutions and indel-Type off-target effects.

A

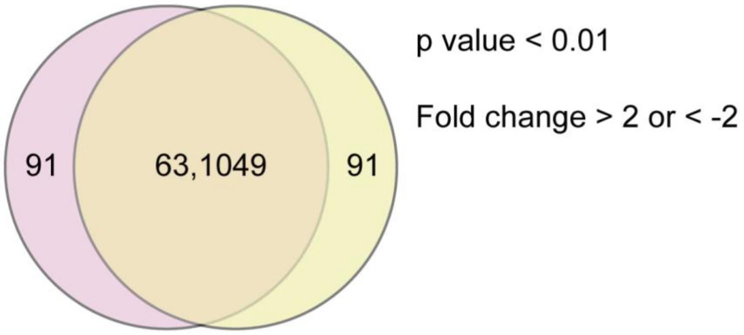

B

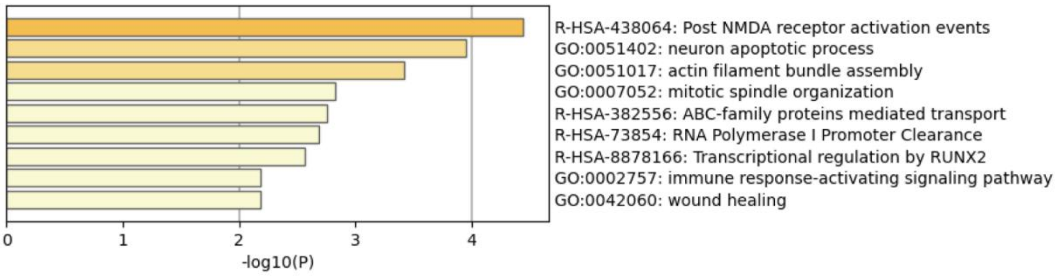

Supplementary Figure 16. Differentially expressed genes and pathway analysis in mCherry reporter Cells transfected with epiPE2 for 10 Days. A. Venn diagram showing differentially expressed genes in mCherry reporter cells edited with epiPE2 for 10 days compared to untreated mCherry reporter cells. B. Pathway analysis of differentially expressed genes in mCherry reporter cells edited with epiPE2 for 10 days compared to untreated mCherry reporter cells.

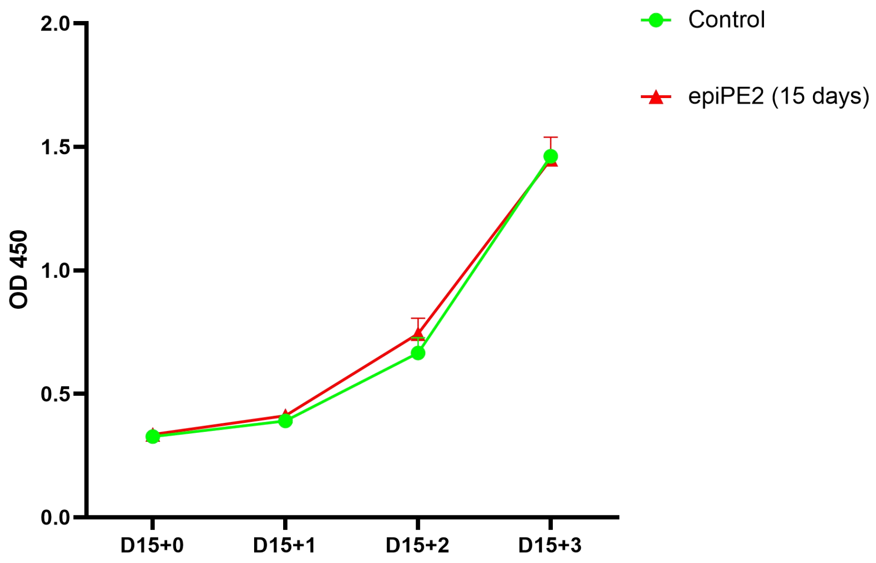

Supplementary Figure 17. Comparison of cell viability between mCherry reporter cells edited with epiPE2 for 15 days and untreated mCherry reporter cells.

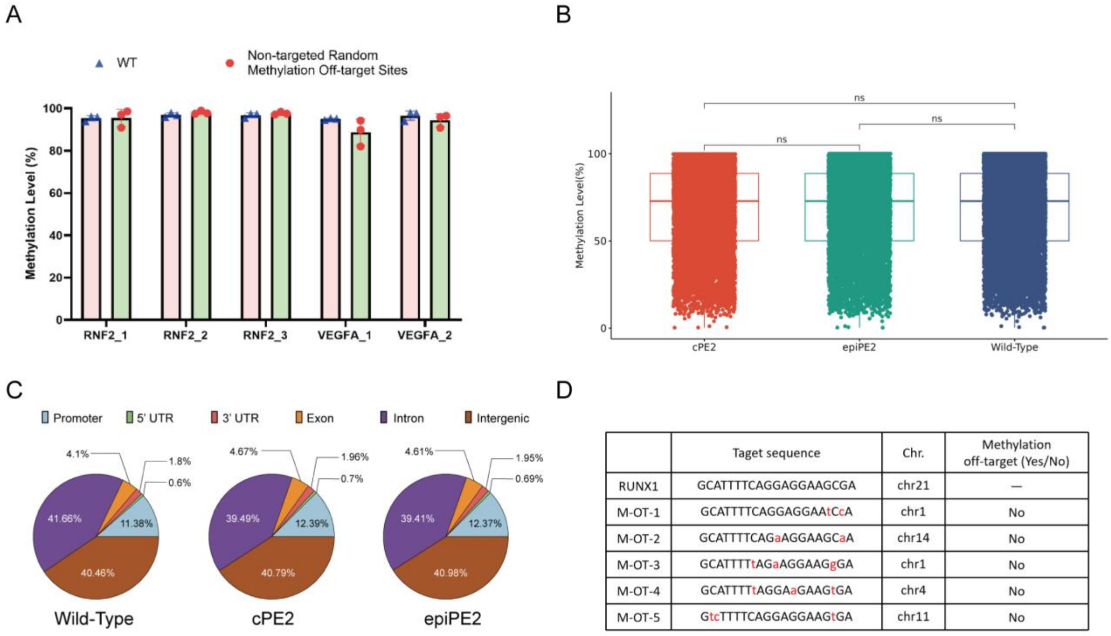

	Taget sequence	Chr.	Methylation off-target (Yes/No)
RUNX1	GCATTTTCAGGAGGAAGCGA	chr21	—
M-OT-1	GCATTTTCAGGAGGAAtCcA	chr1	No
M-OT-2	GCATTTTCAGaAGGAAGCaA	chr14	No
M-OT-3	GCATTTTtAGaAGGAAGgGA	chr1	No
M-OT-4	GCATTTTtAGGAaGAAGtGA	chr4	No
M-OT-5	GtcTTTTCAGGAGGAAGtGA	chr11	No

Supplementary Figure 18. Methylation profiling of epiPE2-treated cells: off-target effects and genome-wide distribution. A. Methylation levels between the WT group cells and epiPE2-treated cells at certain non-targeted sites. B. Distribution of the methylation levels in the common regions (T-test). C. Statistical plot depicting the genome-wide distribution annotation of methylated regions. D. Methylation off-target site analysis. Red letters represent mismatched bases.

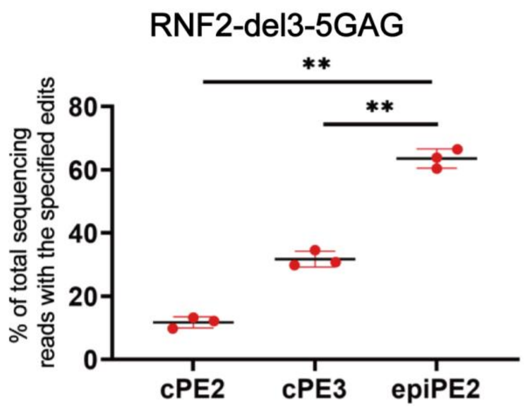

Supplementary Figure 19. Estimated editing efficiencies frequencies induced by cPE2, cPE3 and epiPE2. Frequencies (mean ± s.e.m.) were calculated from three independent experiments (n = 3). *P* values were obtained using two-tailed Student's t-tests. **P* < 0.05, ***P* < 0.01 and ****P* < 0.001.

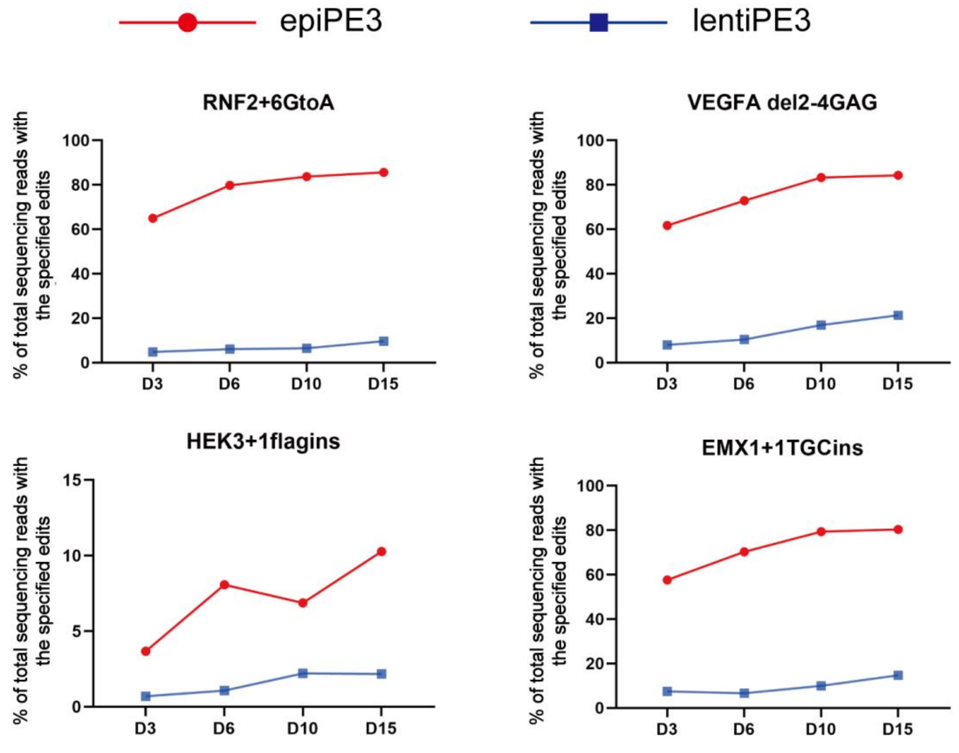

Supplementary Figure 20. Comparison of editing efficiency between epiPE3 and lentiPE3.

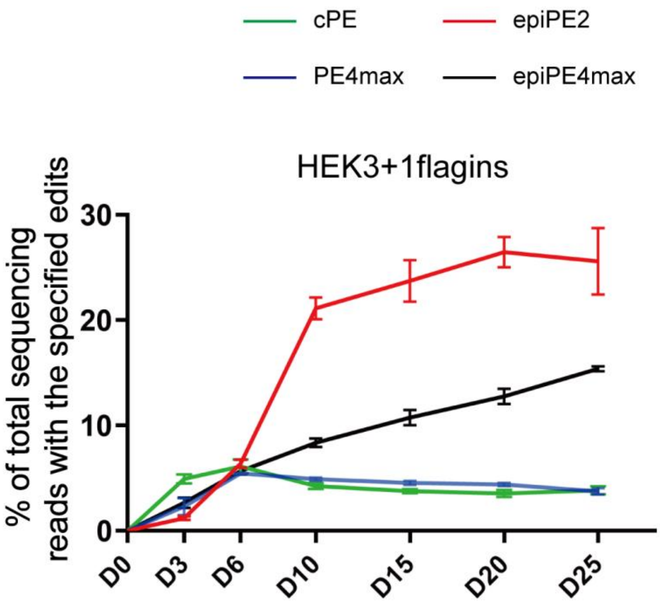

Supplementary Figure 21. Comparison of the efficiency between cPE2, cPE4max, epiPE4max and epiPE2 in different time points. Frequencies (mean ± s.e.m.) were calculated from three independent experiments (n = 3). *P* values were obtained using two-tailed Student's t-tests. **P*< 0.05, ***P*< 0.01 and ****P*< 0.001.

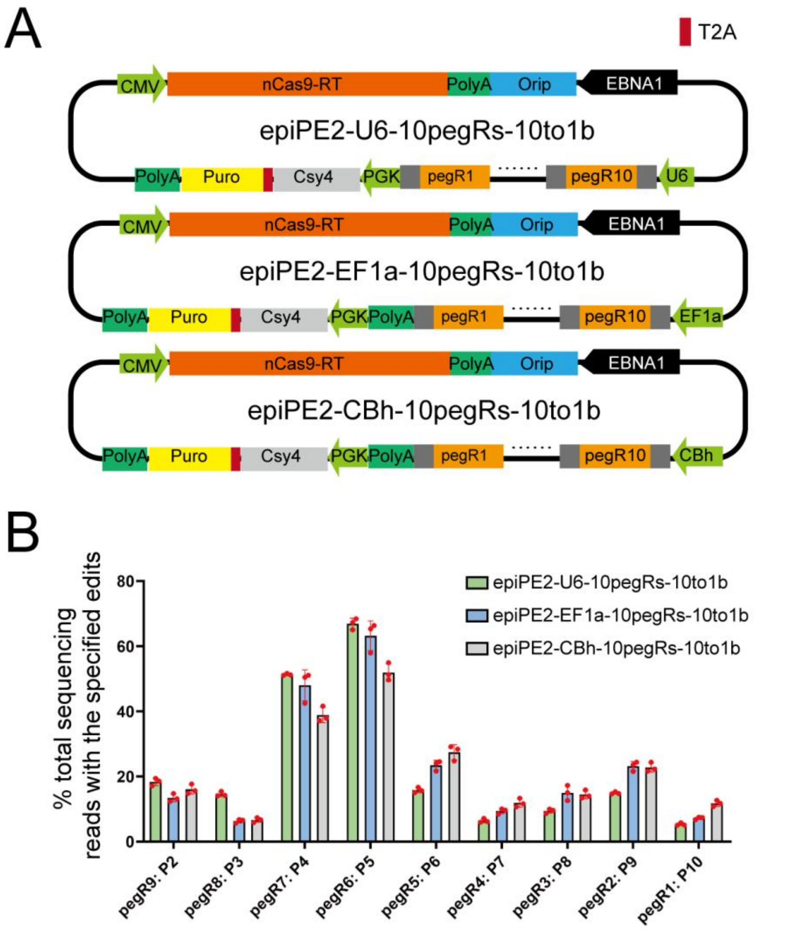

pegR1: RNF2-del3-5GAG	pegR2: EMX1-+5GtoT	pegR3: FANCF-+4GATins
pegR4: RUNX1-del2-4GAG	pegR5: DNMT1-+6GtoC	pegR6: HEK3+1CTTins
pegR7: VEGFA-del2-4GAG	pegR8: PRNP-+6GtoT	pegR9: HEXA-+1TATCins

Supplementary Figure 22. One-step generation multiple precise genome-editing by Csy4-mediated processing system. A. Structure of three constructs for expressing pegRNA 10 to 1. **B.** Mutation frequencies and analysis among three vectors of pegRNA 10 to 1. P2 to p10 indicate the position of pegRNA in the sequences.

epiPE2-U6-3pegRs

	FANCF+5GtoT	VEGFA-+5GtoT	RUNX1-+5GtoT
Day3	<0.2	<0.2	<0.2
Day6	<0.2	<0.2	<0.2
Day10	<0.2	<0.2	<0.2
Day15	<0.2	<0.2	<0.2
Day20	<0.2	<0.2	<0.2
Day25	<0.2	<0.2	<0.2

epiPE2-EF1a-3pegRs

	FANCF+5GtoT	VEGFA-+5GtoT	RUNX1-+5GtoT
Day3	<0.2	<0.2	<0.2
Day6	<0.2	<0.2	<0.2
Day10	<0.2	<0.2	<0.2
Day15	<0.2	<0.2	<0.2
Day20	<0.2	<0.2	<0.2
Day25	<0.2	<0.2	<0.2

10 pegRNAs-containing plasmids

	RNF2-del3-5GAG	EMX1-+5GtoT	FANCF-+4GATins	RUNX1-del2-4GAG	DNMT1-+6GtoC	HEK3+1CTTins	VEGFA-del2-4GAG	PRNP-+6GtoT	HEXA-+1TATCins	CFTR-del15-17CTT
epiPE2-U6-10pegRs-1to10b	<0.2	<0.2	<0.2	<0.2	<0.2	<0.2	<0.2	0.23	<0.2	<0.2
epiPE2-U6-10pegRs-10to1b	<0.2	<0.2	<0.2	<0.2	<0.2	<0.2	0.20	<0.2	<0.2	<0.2
epiPE2-EF1a-10pegRs-10to1b	<0.2	<0.2	<0.2	<0.2	<0.2	<0.2	<0.2	<0.2	<0.2	<0.2
epiPE2-CBh-10pegRs-10to1b	<0.2	<0.2	<0.2	<0.2	<0.2	<0.2	<0.2	<0.2	<0.2	<0.2

Supplementary Figure 23. Off-target analysis of pegRNAs in different plasmids.
